# Peaks, sources, and immediate health impacts of PM_2.5_ and PM_1_ exposure in Indonesia and Taiwan with microsensors

**DOI:** 10.1038/s41370-024-00689-4

**Published:** 2024-05-28

**Authors:** Shih-Chun Candice Lung, Ming-Chien Mark Tsou, Chih-Hui Chloe Cheng, Wiwiek Setyawati

**Affiliations:** 1 Research Center for Environmental Changes, Academia Sinica, Taipei, Taiwan, ROC; 2 Department of Atmospheric Sciences, National Taiwan University, Taipei, Taiwan, ROC; 3 Institute of Environmental and Occupational Health Sciences, National Taiwan University, Taipei, Taiwan, ROC; 4 Research Center for Climate and Atmosphere, National Research and Innovation Agency (BRIN), Kota Bandung, Indonesia

**Keywords:** PM exposure and health, Low-cost sensors, PM sensing device, Asian PM epidemiological panel study, Hi-ASAP

## Abstract

**Background:**

Microsensors have been used for the high-resolution particulate matter (PM) monitoring.

**Objectives:**

This study applies PM and health microsensors with the objective of assessing the peak exposure, sources, and immediate health impacts of PM_2.5_ and PM_1_ in two Asian countries.

**Methods:**

Exposure assessment and health evaluation were carried out for 50 subjects in 2018 and 2019 in Bandung, Indonesia and for 55 subjects in 2019 and 2020 in Kaohsiung, Taiwan. Calibrated AS-LUNG sets and medical-certified RootiRx® sensors were used to assess PM and heart-rate variability (HRV), respectively.

**Results:**

Overall, the 5-min mean exposure of PM_2.5_ and PM_1_ was 30.4 ± 20.0 and 27.0 ± 15.7 µg/m^3^ in Indonesia and 14.9 ± 11.2 and 13.9 ± 9.8 µg/m^3^ in Taiwan, respectively. The maximum 5-min peak PM_2.5_ and PM_1_ exposures were 473.6 and 154.0 µg/m^3^ in Indonesia and 467.4 and 217.7 µg/m^3^ in Taiwan, respectively. Community factories and mosquito coil burning are the two most important exposure sources, resulting in, on average, 4.73 and 5.82 µg/m^3^ higher PM_2.5_ exposure increments for Indonesian subjects and 10.1 and 9.82 µg/m^3^ higher PM_2.5_ exposure for Taiwanese subjects compared to non-exposure periods, respectively. Moreover, agricultural waste burning and incense burning were another two important exposure sources, but only in Taiwan. Furthermore, 5-min PM_2.5_ and PM_1_ exposure had statistically significantly immediate impacts on the HRV indices and heart rates of all subjects in Taiwan and the scooter subjects in Indonesia with generalized additive mixed models. The HRV change for a 10 µg/m^3^ increase in PM_2.5_ and PM_1_ ranged from −0.9% to −2.5% except for ratio of low-high frequency, with greater impacts associated with PM_1_ than PM_2.5_ in both countries.

**Impact statement:**

This work highlights the ability of microsensors to capture high peaks of PM_2.5_ and PM_1_, to identify exposure sources through the integration of activity records, and to assess immediate changes in heart rate variability for a panel of approximately 50 subjects in Indonesia and Taiwan. This study stands out as one of the few to demonstrate the immediate health impacts of peak PM, complementing to the short-term (days or weeks) or long-term effects (months or longer) assessed in most epidemiological studies. The technology/methodology employed offer great potential for researchers in the resource-limited countries with high PM_2.5_ and PM_1_ levels.

## Introduction

The application of microsensors has rapidly increased in environmental research due to their low cost and ease of use. The known accuracy limitation issue was resolved by calibrating with research-grade instruments [1]. The sensing observations provide a much higher spatiotemporal resolution of pollutant levels than typical hourly measurements from worldwide environmental agencies. Microsensors can complement regulatory monitoring in locations without standard monitoring stations and can also be used for personal exposure assessment [2]. The current study further takes advantage of the advancements in microsensors in medical science on top of those in environmental research to assess the exposure and health impacts of particulate matter with aerodynamic diameter less than or equal to 2.5 μm (PM_2.5_) and PM_1_ in two Asian countries.

Exposure to ambient PM is the largest environmental risk factor for health [3]; the most concerning component is PM_2.5_. The top 15 capital cities in the world in 2021 with the worst PM_2.5_ levels were all in low- and middle-income countries, with annual means higher than 35 μg/m^3^ [4], seven times higher than the 2021 annual guideline recommended by the World Health Organization (WHO), 5 μg/m^3^ [5]. PM_2.5_ is a classified human carcinogen [6]. Long-term exposure to high concentrations of PM_2.5_ increases the risk of respiratory, cardiovascular, and all-cause mortality [7–9]. Around 4.1 million premature deaths worldwide in 2019 were attributable to PM_2.5_ [10]. The damage coefficients of the PM_2.5_–health relationship can be more accurately estimated with more spatially resolved exposure estimates [11], which can be provided by microsensors.

Moreover, short-term PM_2.5_ exposure can cause the exacerbation of respiratory diseases and changes in heart rate variability (HRV) [12, 13]. PM_2.5_ peaks may also be responsible for stroke [14]. Lightweight PM_2.5_ microsensors with a high temporal resolution are good tools in quantifying peak exposure, which has been difficult to capture with regulatory monitoring. Together with source records, exposure sources responsible for those peaks can be identified. Targeted source controls in residential communities can lower peak exposure and the associated health risks for residents.

In addition, studies have found that smaller PM such as PM_1_ may penetrate deeper into the respiratory tract, leading to greater toxicity [15]. Three recent publications reviewed the health impacts of PM_1_ with a systematic review and meta-analysis. They concluded that significant associations were found between short-term or long-term PM_1_ exposure and the mortality of cardiovascular/respiratory systems and respiratory morbidity for children, adolescents, and adults [16–18]. They also suggested that more research needs to be carried out in different regions to assess the health impacts of PM_1_. New microsensors provide opportunities to fill this gap.

Furthermore, microsensors with medical certifications were applied to assess the health impacts of peak PM_2.5_ in our earlier study with HRV as a health outcome measure [19]. HRV is a marker of cardiac autonomic balance [20]; reduced HRV has been associated with an increased risk of myocardial infarction [21]. Epidemiological studies have demonstrated that PM_2.5_ exposure is associated with reduced HRV [22–26]. The 2010 American Heart Association provided a comprehensive review on PM exposure and cardiovascular disease, outlining three biological pathways. The first involves the release of proinflammatory mediators or vasculoactive molecules from lung-based cells. The second pathway is the perturbation of systemic autonomic nervous system (ANS) balance or heart rhythm by particle interactions with lung receptors or nerves. And the third one is the translocation of ultra-fine particles or particle constituents into the systemic circulation [27]. The hyperacute physiological responses occurred minutes to hours after PM exposure are likely mediated through the second and the third pathways [27]. Recent developments of light-weight and easily-carried biosensors offer a way to measure HRV changes which are signals of the ANS changes (the second pathway). Most of the previous studies used traditional Holter monitors, which are heavy with 12-lead patches; thus, the monitors increase the burden of the subjects and may interfere with their daily routines. A lightweight medically-certified HRV microsensor, with much less burden on the subjects [19, 28], was applied in the current study to assess the immediate HRV impacts associated with PM exposure.

The current study takes advantage of the recent advances in microsensors in both the environment and health fields with the objective of assessing peak exposure levels, sources, and the immediate health impacts of PM_2.5_ and PM_1_ in two Asian countries, Indonesia, and Taiwan. To apply microsensors in assessing PM and health issues in Asia is particularly important. First, most Asian capital cities have much higher annual PM_2.5_ means (measured by monitoring stations) than the WHO guideline [4]. Secondly, due to their proximity to PM sources and resulting higher exposure levels [29–32], Asian urban dwellers face significantly greater risks from PM than current estimates based on regulatory measurements. Thirdly, the high population density in Asian cities aggravates the population’s PM health risks. Fourthly, most Asian countries are in the developing stage, without much funding for research; low-cost PM microsensors are thus affordable tools for PM and health research in this region.

This study is one of the few to apply research-grade low-cost environmental microsensors and medically-certified health microsensors in epidemiological panel studies. To facilitate microsensor application in PM and health research in Asia, an initiative called the Health Investigation and Air Sensing for Asian Pollution (Hi-ASAP) was established, with a series of training workshops for technology/methodology transfer. Hi-ASAP, consisting of researchers from nine countries, was developed under the umbrella of the International Global Atmospheric Chemistry Project–Monsoon Asia and Oceania Networking Group [33]. The current study represents one of the Hi-ASAP efforts to assess the exposure sources and health impacts of PM in Asia, using a consistent methodology. This work highlights the ability of microsensors to capture peaks of PM_2.5_ and PM_1_, the identification of exposure sources through the integration of PM levels with activity records, and the immediate impacts observed in HRV indices among a panel of approximately 50 subjects in both countries. It provides a good demonstration of applying microsensors in quantifying peak exposure as well as the contribution of exposure sources and the HRV impacts of PM_2.5_ and PM_1_ exposures. The exposure sources identified in this work can serve as a primer for scientists in other Asian countries with similar cultures and living styles to investigate PM sources in their own communities. Prioritized control strategies targeting the responsible sources can be implemented afterwards to reduce the chances of short-term peak PM exposures, which may lead to lethal health impacts such as stroke.

## Materials and methods

Two panels were recruited in this study, one in Bandung, Indonesia, and one in Kaohsiung, Taiwan. Bandung (6°54′43″S, 107°36′35″E) is the capital city of the Indonesian province of West Java. The Bandung Basin Metropolitan Area is the country’s third-largest metropolitan area, with over 8 million inhabitants. Kaohsiung city (22°36′54″N, 120°17′51″E) is the largest city in southern Taiwan, with ~2.73 million residents. Subject recruitment, microsensors, exposure assessment and health evaluation, and data analysis are described below.

### Subject recruitment

The Indonesian recruitment was conducted in Bandung in 2018 and 2019 with flyers distributed around an institute, following the inclusion criteria: (1) aged 18–65 years, (2) with more than 1 h of daily commuting time, (3) not a current smoker or an alcohol drinker, and (4) without physician-diagnosed cardiopulmonary diseases and medication that may affect cardiopulmonary function and sleep. Personal exposure and health evaluation were carried out from November 2018 to January 2019 (wet season) and from July to September 2019 (dry season). Thirty-two participants were recruited for monitoring in the wet season, but only 14 of them were available for dry-season monitoring. Thus, another 18 subjects were recruited, bringing the total number of subjects to 50. Part of the research findings has been published in another paper [34].

The Taiwanese recruitment was carried out in Kaohsiung in 2019 and 2020 via flyer distribution and recruitment meetings with slightly different inclusion criteria compared to those in Indonesia. The criteria were (1) aged 40–75 living in the studied community, (2) a non-smoker or quit smoking for more than 1 year, and (3) without history of hypertension or heart-related diseases. The studied community was randomly selected from the entire Kaohsiung metropolitan, according to the strategy of 2010 Taiwan Social Change Survey described in ref. [35]. In brief, all townships in Taiwan were classified according to the geographical locations and urbanization levels. Random sampling was employed to select the townships and subsequently communities. Personal exposure and health evaluations were conducted from July to August 2019 (summer) and from February to March 2020 (winter). Fifty participants were recruited for monitoring in summer, but five of them dropped out for winter monitoring. Therefore, another five subjects were recruited for winter, bringing the total number of subjects to 55.

### Microsensors: AS-LUNG-P, AS-LUNG-O, and Rooti

AS-LUNG-P sets (portable-type of Acadmia Sinica-LUNG, AS-LUNG) were used to monitor the PM_2.5_ and PM_1_ exposure; these have been used in our earlier study [19]. AS-LUNG-P is a personal device equipped with sensors that detect PM_2.5_ and PM_1_ (PMS3003, Plantower, Beijing, China), temperature/humidity (SHT31, Sensirion AG, Switzerland), location (GPS, u-blox, Switzerland), and motion (ADXL345B, Analog Devices, Inc., USA) [28]. Briefly, it is a small (135 × 70 × 40 mm) and lightweight (153 g) device with an SD card. The sampling frequency was chosen as 15-s in this study. Before monitoring, every AS-LUNG-P device was calibrated with research-grade instruments, GRIMM Model 1.109 or EDM-180 (GRIMM Aerosol Technik Ainring GmbH & Co, Ainring, Germany), in a customized chamber with the procedures described earlier [36]. EDM-180 is a PM_2.5_ Federal Equivalent Method instrument. The obtained correction equations had an *R*^2^ of 0.974-0.995 for PM_2.5_ and 0.981–0.999 for PM_1_. All PM concentrations were transformed into research-grade observations using correction equations.

AS-LUNG-O sets (outdoor-type of AS-LUNG) powered by solar panels were used for ambient PM_2.5_ and PM_1_ monitoring in 1-min resolution in both cities, with the same sensors as AS-LUNG-P integrated inside a waterproof casing [31]. In Indonesia, one AS-LUNG-O was installed on the rooftop of a four-story building in downtown Bandung. The collected data represented the ambient PM levels since most subjects lived within 10 km of this building. The ambient PM_1_ data in Indonesia were lost and therefore were not included here. In Taiwan, one AS-LUNG-O was set-up at third-floor height within the studied community where the recruited subjects lived. AS-LUNG-O sets were calibrated as AS-LUNG-P.

HRV monitoring was carried out with RootiRx® (shorten as Rooti), which is a portable single-lead electrocardiogram event recorder (RootiCare®, Rooti Labs Ltd, Taipei, Taiwan) certified as a medical device by the EU, US, and Taiwan (www.rooticare.com). Rooti was evaluated to have an average beat per minute correlation of 0.98 with a standard 12-lead Holter monitor [37]. It is a water-resistant, compact, small (62 × 22.5 × 8.45 mm) and lightweight (14 ± 1 g) patch with a sampling rate of 500 Hz. The data were analyzed using Rooti Labs Ltd.’s proprietary algorithms. The study assessed two time-domain indices (standard deviation of normal to normal intervals (SDNN) and root mean square of successive differences between normal heartbeats (RMSSD)) and five frequency-domain indices (high-frequency (HF, 0.15–0.4 Hz), low-frequency (LF, 0.04–0.15 Hz), very-low-frequency (VLF, 0.003–0.04 Hz), total power (TP), and ratio of LF to HF power (LF/HF)). Heart rate (HR) was also assessed. To reduce bias caused by artifacts, a noise filter was defined as the mean SDNN of >400 ms and the mean HR of <40 beats/min and >200 beats/min in a single 5-min segment.

### Exposure assessment and health evaluation

Written informed consent was obtained from all subjects prior to monitoring. Interviews were conducted by trained interviewers to gather their basic characteristics. All subjects were asked to carry one AS-LUNG-P and one Rooti for PM and HRV monitoring, respectively. They were asked to maintain their daily routines as usual. The AS-LUNG-P needed to be carried at all times except during showering (placed outside bathroom) and sleeping (placed on nearby nightstands), while Rooti needed to be worn at all times. In Indonesia, the subjects carried AS-LUNG-P for one to six 24-h periods, and Rooti for one to three 24-h periods, respectively, on working days. In Taiwan, the subjects were asked to carry AS-LUNG-P for seven 24-h periods and Rooti for one 48-h period, without excluding non-working days. Some subjects wore Rooti for a second 48-h period due to a high missing rate (>20%) in the first 48-h period. All subjects were also asked to maintain time–activity diaries (TADs) documenting their activities, locations, ventilation status if indoors, and a maximum of two air pollution sources they encountered for every 30-min interval. Only visible nearby sources were recorded. For example, actual exposure to emissions from community factories was recorded rather than recording speculative exposure to emissions from far-away sources. In Indonesia, TADs were not filled out at strictly 30-min intervals, but were checked to ensure important activities such as commuting and encountered sources were recorded. In Taiwan, each subject’s TADs were checked daily by a trained interviewer.

### Data analysis

Data cleaning was performed by removing PM measurements less than 1 μg/m^3^ and ghost peaks, defined as instances where a 15-s peak exceeded ten times the means of the 5 readings before and after the peak. Afterwards, hours with rainfall were excluded. Since HRV indices are typically analyzed with a 5-min resolution, 15-s PM measurements within each 5-min interval were averaged to match HRV measurements. If missing data comprised more than half of a 5-min interval, all data within that interval were removed. The data missing rate was 4.4% and 1.9% in Indonesia and Taiwan, respectively. Source contributions to PM were assessed by using a generalized additive mixed model (GAMM) to analyze the relationship between PM and TAD records. GAMM was chosen because the PM observations were repeated measurements that may have been correlated within the same individual and may have had a nonlinear relationship with independent variables [31]. The model is shown below:

1 $${{{{{{\rm{PM}}}}}}}_{{{{{{\rm{Personal}}}}}}}-{{{{{{\rm{PM}}}}}}}_{{{{{{\rm{ambient}}}}}}}={\alpha }_{0}+\sum \beta {iXi}+{{{{\mathrm{\zeta \; Season}}}}}+{f}_{1}\left({{{{{\rm{humidity}}}}}}\right)+{{{{{{\rm{\gamma }}}}}}}_{{{{{{\rm{Subject}}}}}}}+\varepsilon$$

where PM_personal_ is the 5-min personal PM_2.5_ or PM_1_ exposure and PM_ambient_ is the corresponding ambient 5-min PM level obtained by AS-LUNG-O; ${{{{{{\rm{\alpha }}}}}}}_{0}$ is the intercept; βi is the regression coefficient of Xi, which is a dummy variable representing different sources recorded in TADs; γ is the random effect of subjects; and *ε* is the error term. The adjusting factors include season (Indonesia is wet/dry; Taiwan is summer/winter) and relative humidity. The thin plate spline function (${f}_{1}$) was used to control for the nonlinear fixed effect of relative humidity. Since temperature and humidity were highly correlated, the models were adjusted for humidity rather than temperature to avoid collinearity. A continuous first-order autoregressive correlation structure for time was considered.

Periods when the subjects were outside of the studied city were excluded because our sensors did not measure ambient levels in those locations. Sources that were recorded less than ten times were not included in order to focus on significant ones; and only the primary source in one 30-min period was put into the model, which was defined as the source affecting the subject with the longest duration in this 30-min interval. The sources included were agricultural waste burning, aromatic products (such as candle burning for a soothing effect, hair spray, etc.), cleaning, cooking, environmental tobacco smoke (ETS), community factories, garbage burning, incense burning, mosquito coil burning, and traffic emission. Exposure to traffic emissions was further classified according to the transportation modes used by the subjects, namely walking, biking, scootering, driving a car, and taking a bus (or minibus). Dummy variables were employed, with the exposure levels in non-commuting periods as the reference. For consistency of time resolution with PM and HRV measurements, TADs were reprocessed and reported in 5-min resolution hereafter.

For PM–health evaluation, all HRV indices and HR were log10-transformed to meet normality assumptions. A GAMM was applied to assess the associations between PM levels and HRV, excluding those observations during sleep, as below:

2 $$\log \left({{{{{\rm{HRV}}}}}}\right)= 	 {{{{{{\rm{\beta }}}}}}}_{0}+{{{{{{\rm{\beta }}}}}}}_{1}{{{{{{\rm{PM}}}}}}}_{{{{{{\rm{personal}}}}}}}+{{{{{{\rm{\beta }}}}}}}_{2}{{{{{\rm{Age}}}}}}+{{{{{{\rm{\beta }}}}}}}_{3}{{\mbox{Gender}}}+{{{{{{\rm{\beta }}}}}}}_{4}{{{{{\rm{BMI}}}}}}+{{{{{{\rm{\beta }}}}}}}_{5}{{\mbox{Outdoor}}} \\ 	 +{{{{{{\rm{\beta }}}}}}}_{6}{{{{{\rm{Season}}}}}}+{{f}_{1}\left({{{{{\rm{Activity}}}}}}\right)+{f}_{1}\left({{{{{\rm{humidity}}}}}}\right)+{f}_{2}\left({{{{{\rm{Time}}}}}}\right)+\gamma }_{{{{{{\rm{subject}}}}}}}+\varepsilon$$

where βi is the regression coefficient of explanatory variables. Known factors affecting HRV were adjusted in the model, including age, gender, body mass index (BMI), and activity level [19, 38–40]. Age was classified as <40 (set as 0) and ≥40 in Indonesia, and <65 and ≥65 in Taiwan. Gender was coded as 0 for females and 1 for males. BMI was classified as low (set as 0) and high according to the respective definition in each country; high BMI was defined as >25 kg/m^2^ in Indonesia [41] and ≥24 kg/m^2^ in Taiwan [42]. Outdoor status was assigned a value of 0 for indoors and 1 for outdoors. Season was coded as 0 for wet and 1 for dry in Indonesia and 0 for summer and 1 for winter in Taiwan. Individual differences were accounted for with a random effect. The thin plate spline function (${f}_{1}$) was used to control for the nonlinear fixed effects of activity and humidity, while the penalized cubic regression spline function (${f}_{2}$) was used to adjust for the time of day. Activity intensity was calculated as the maximum acceleration in each of the three dimensions in every 5-min epoch using the following equation: activity intensity =$\sqrt{{x}^{2}+{y}^{2}+{z}^{2}}$, where X, Y, and Z represent the maximum acceleration in the left–right, cranial–caudal, and dorsal–ventral dimensions, respectively. A continuous first-order autoregressive correlation structure for time was considered. The lag effects on HRV were also assessed with GAMM [19]. Since only data during non-sleeping periods were used, the lag effects were assessed for only up to 6 h. Data analysis was performed using RStudio version 1.1.456 (RStudio, USA, 2018) and R Version 4.0.2 (The R Foundation, Austria, 2020).

## Results and discussion

### PM_2.5_ and PM_1_ exposure levels of the subjects in two countries

Table 1 shows the characteristics of the subjects. After data cleaning, there were only 49 Indonesian and 51 Taiwanese subjects left in the final dataset. Of these Indonesian subjects, 28 were males and 21 females, and 28 of them were under the age of 40. In Taiwan, there were 19 males and 32 females, with 28 of them under the age of 65. Subjects in both countries included white-collar workers, blue-collar workers, and housewife/unemployed. There were 20 and 29 subjects in the high-BMI group in Indonesia and Taiwan for health evaluation, respectively.

**Table 1:** Characteristics of the subjects in Indonesia and Taiwan.

Indonesia				Taiwan			
Characteristics	*n*				*n*		
	All	Wet	Dry		All	Summer	Winter
Gender							
Male	28	16	19	Male	19	16	19
Female	21	16	12	Female	32	30	28
Age							
<40	28	16	15	<65	28	27	25
≥40	21	16	16	≥65	23	19	22
BMI							
≤25	29	18	19	<24	22	18	19
>25	20	14	12	≥24	29	28	28
Occupations							
White collar	38	28	23	White collar	9	8	8
Blue-collar	6	2	4	Blue-collar	23	19	22
Housekeeper/Unemployed	3	1	3	Housekeeper/Unemployed	19	19	17
Unknown	2	1	1	Unknown	0	0	0

Table 2 shows the 5-min resolution of PM_2.5_, PM_1_ and PM_1_/PM_2.5_ ratios in both countries; the data can be found in [43]. Overall, the mean personal exposure of PM_2.5_ and PM_1_ was 30.4 ± 20.0 and 27.0 ± 15.7 µg/m^3^ in Indonesia and 14.9 ± 11.2 and 13.9 ± 9.8 µg/m^3^ in Taiwan, respectively. The means and standard deviations of PM_2.5_ and PM_1_ in Indonesia did not differ much in both seasons, with the maximum level of PM_2.5_ (473.6 µg/m^3^, during exposure to cooking) occurring in the wet season and that of PM_1_ (154.0 µg/m^3^, during exposure to mosquito coil burning) occurring in the dry season. In contrast, in Taiwan, the mean levels of PM_2.5_ and PM_1_ in winter were more than twice those in summer, with the maximum level of PM_2.5_ (467.4 µg/m^3^) being observed in summer and that of PM_1_ (217.7 µg/m^3^) being observed in winter, both occurring during exposure to emissions from community factories. PM_2.5_ and PM_1_ exposure in Taiwan was generally lower than that in Indonesia. The PM_1_/PM_2.5_ ratios of personal exposure were, on average, 0.90 in Indonesia and 0.92 in Taiwan, showing that the majority of PM_2.5_ is PM_1_. Additionally, these ratios did not change much in different seasons.

**Table 2:** Mean and standard deviation (SD, in parentheses) of personal and ambient levels of 5-min PM_2.5_, PM_1_ and PM_1_/PM_2.5_ ratios in (a) Indonesia and (b) Taiwan.

(a) Indonesia							
Type	Season	*n*	PM_2.5_ (μg/m^3^)		PM_1_ (μg/m^3^)		Mean (SD) of PM_1_/PM_2.5_ ratio
			Mean (SD)	Max	Mean (SD)	Max	
Personal exposure	All	32,066	30.4 (20.0)	473.6	27.0 (15.7)	154.0	0.9 (0.04)
	Wet	18,206	30.0 (23.1)	473.6	26.5 (17.6)	153.4	0.9 (0.04)
	Dry	13,860	30.9 (15.0)	301.6	27.6 (12.8)	154.0	0.9 (0.03)
Ambient level	All	32,066	32.0 (14.0)	98.6	--^a^	--	--
	Wet	18,206	29.4 (11.6)	77.3	--	--	--
	Dry	13,860	35.3 (16.0)	98.6	--	--	--
**(b) Taiwan**							
Personal exposure	All	51,752	14.9 (11.2)	467.4	13.9 (9.8)	217.7	0.92 (0.07)
	Summer	25,926	8.9 (8.8)	467.4	8.2 (6.1)	120.9	0.92 (0.07)
	Winter	25,826	21.0 (10.0)	361.3	19.6 (9.5)	217.7	0.92 (0.07)
Ambient level	All	51,752	21.2 (8.1)	59.7	20.5 (7.4)	50.7	0.97 (0.03)
	Summer	25,926	21.0 (7.6)	51.9	20.9 (7.5)	50.7	1.0 (0.01)
	Winter	25,826	21.5 (8.5)	59.7	20.2 (7.3)	49.3	0.95 (0.04)

^a^Data were lost.

In ambient air, the overall PM_2.5_ level in Indonesia was 32.0 ± 14.0 µg/m^3^. That in the dry season was slightly higher than the personal exposure, and that in the wet season was in a similar range. However, the maximum PM_2.5_ in ambient air was 98.6 µg/m^3^, about one-quarter of the highest level of personal exposure (473.6 µg/m^3^). In Taiwan, the mean ambient PM_2.5_ and PM_1_ levels were 21.2 ± 8.1 and 20.5 ± 7.4 µg/m^3^, respectively, and they were slightly higher than the personal exposure in winter and almost double that in summer. Nevertheless, the maximum PM_2.5_ level in ambient air was 59.7 µg/m^3^ in Taiwan, about one-eighth of the highest personal exposure level (467.4 µg/m^3^). A huge discrepancy between personal peak PM exposure and maximum ambient levels was observed for both countries, presumably due to close human contact with nearby sources, again emphasizing the importance of assessing actual personal exposure in exposure–health evaluations.

With microsensors taking high-resolution measurements, peak exposure can be captured. Figure S1 (in Supplementary Information) shows one example of personal PM_2.5_ exposure in each country riding scooters with a 1-min resolution. In the morning, the highest peak PM_2.5_ of the Indonesian subject was 95.7 µg/m^3^, two times higher than the mean levels (37.0 ± 16.0 µg/m^3^) during the 1-h commuting period; the mean PM_2.5_ in the evening commuting period was 22.9 ± 10.0 µg/m^3^, with a peak of 70.6 µg/m^3^. In Taiwan, the subject was exposed to peak levels of 19.6 and 21.8 µg/m^3^, about 30% higher than the mean levels of 15.2 ± 3.1 and 15.7 ± 2.4 µg/m^3^ during the morning and evening commute, respectively.

Most epidemiological studies using ambient levels from standard monitoring stations with hourly means as surrogates for exposure [7–9] lack information on peak values. For tracking pollution trends, peak values may not be suitable for regulatory purposes. Nevertheless, for environmental health research, peak exposure levels are important since they could be responsible for asthmatic attacks and strokes [14, 44]. With the assistance of lightweight personal microsensors with a high temporal resolution, peak exposure in minutes can be captured, providing valuable information for exposure assessment and exposure–health evaluation.

Table 3 shows the means and standard deviations of seven HRV indices and HR during non-sleeping periods. Since the physiological signals during sleeping and non-sleeping periods are very different, only observations during non-sleeping periods were used for PM-health evaluation. There was no significant difference between two seasons in both countries. Most of the means and standard deviations of these indices of Taiwanese subjects were smaller than those of Indonesian subjects, presumably due to the older subjects in Taiwan.

**Table 3:** Mean and standard deviation (SD, in parentheses) of 5-min heart rate variability (HRV) indices and heart rate (HR) during non-sleeping period in (a) Indonesia and (b) Taiwan.

(a) Indonesia									
Season	SDNN (ms)	RMSSD (ms)	LF/HF	HF (ms^2^)	LF (ms^2^)	VLF (ms^2^)	TP (ms^2^)	HR (bpm)	n
All	53.8 (28.8)	26.9 (16.5)	3.9 (2.8)	428.4 (641.8)	1099.2 (1464.5)	2495.3 (3334.9)	4110.3 (4995.3)	83.9 (13.6)	21519
Wet	55.1 (29.1)	27.4 (17.0)	4.1 (3.0)	430.4 (680.7)	1100.6 (1511.7)	2603.2 (3423.3)	4225.9 (5129.9)	82.1 (12.9)	12489
Dry	51.9 (28.3)	26.2 (15.7)	3.6 (2.5)	425.5 (583.8)	1097.4 (1396.6)	2346.1 (3202.8)	3950.3 (4798.6)	86.5 (14.1)	9030
**(b) Taiwan**									
All	42.8 (23.5)	20.0 (12.7)	3.8 (3.0)	240.4 (501.7)	570.1 (980.9)	1580.0 (2146.8)	2444.6 (3244.8)	79.1 (12.5)	33125
Summer	43.2 (23.5)	20.9 (13.1)	3.6 (2.9)	245.0 (471.2)	554.6 (835.0)	1554.7 (2026.7)	2410.7 (2949.9)	78.2 (12.4)	15782
Winter	42.4 (23.4)	19.1 (12.4)	4.0 (3.2)	236.3 (528.0)	584.3 (1096.8)	1603.0 (2250.4)	2475.4 (3491.3)	80.0 (12.5)	17343

### Exposure sources in two countries

With TADs, it is possible to differentiate the PM levels during exposure to different sources. The frequency of these exposure sources encountered by the subjects is shown in Fig. S2. In both countries, traffic exposure was the most common, and thus was classified into five transportation modes. Among these modes, cars and scooters were the two transportation modes used most frequently by Indonesian subjects; while for Taiwanese subjects, a scooter was the most commonly used mode. Besides traffic emissions, the most common sources in Indonesia and Taiwan were aromatic products and cooking, respectively. Three exposure sources only occurred in Taiwan, namely agricultural waste burning, garbage burning, and incense burning.

**Fig. 1:**
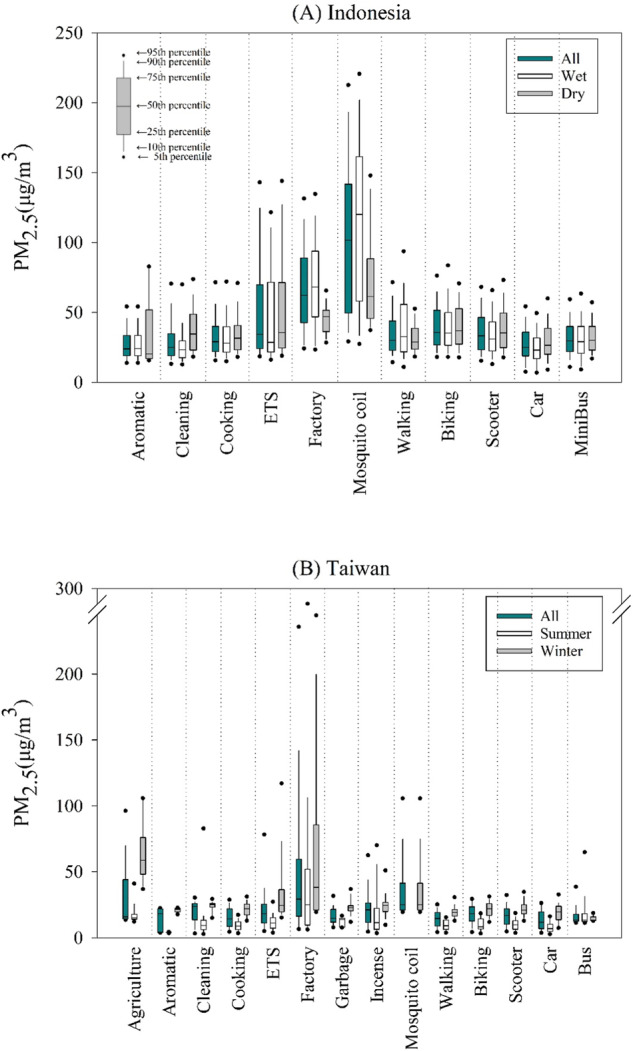
The distributions of the 5-min PM_2.5_ concentrations for different exposure sources or transportation modes. (**a**) Indonesia and (**b**) Taiwan.

Figure 1 shows 5-min PM_2.5_ exposure levels when the subjects were exposed to different sources. Overall, the medians and 90th percentiles of PM_2.5_ exposure levels in Indonesia were higher than those in Taiwan for the same source; nevertheless, Taiwanese subjects were exposed to extremely high levels associated with community factory emissions. Mosquito coil burning and factory emissions are associated with the highest medians and 95th percentiles of PM_2.5_ among all sources in Indonesia and Taiwan, respectively. The PM_1_/PM_2.5_ ratios associated with different sources are shown in Fig. S3. Mosquito coil burning and community factories are associated with the lowest 5th percentiles of PM_1_/PM_2.5_ ratios of all sources in Indonesia and Taiwan, respectively. Additionally, most sources in Indonesia are associated with lower 95th percentiles of PM_1_/PM_2.5_ ratios than those sources in Taiwan. Nevertheless, most of the PM_1_/PM_2.5_ ratios range from 0.7 to 1.0 and do not significantly differ among most sources.

The maximum 5-min PM_2.5_ exposure levels associated with different sources are listed in Table S1. The top two maximums were associated with cooking and mosquito coil burning in Indonesia and with community factories and mosquito coil burning in Taiwan. The ratio of the maximum 5-min PM_2.5_ exposure to the mean exposure of that hour was also calculated. The top two highest ratios in Indonesia are associated with cooking (ratio = 4.9) and mosquito coil burning (3.5), and those in Taiwan are associated with traffic exposure during scootering (3.6) and incense burning (3.4). Moreover, the exposure percentage of that source (considering the exposure duration of the same source) accounted for the overall 24-h PM_2.5_ exposure was calculated. In Indonesia, the top two are associated with aromatic products and mosquito coil burning, which accounted for 42.0% and 18.0% of the 24-h exposure, respectively. In Taiwan, the top two sources were ETS (39.8%) and factory emissions (37.1%). To reduce health risks, sources associated with high maximum/mean ratios or those accounting for a high percentage of daily exposure should be given high priority for source control.

The above source evaluation did not exclude the influence of concurrent ambient levels. Thus, GAMM was further applied to assess the average contribution of different sources to PM_2.5_ and PM_1_ exposure excluding the influence of ambient levels, with results shown in Table 4. There were seasonal differences, with more sources having statistically significant incremental contributions during the dry season in Indonesia. Overall, for Indonesian subjects, cleaning, cooking, community factories, mosquito coil burning, and traffic were statistically significant sources of exposure when observations from both seasons were pooled. Mosquito coil burning and community factories were the top two sources contributing, on average, 5.82 and 4.73 µg/m^3^ for PM_2.5_, respectively (Table 4a). In addition, the PM_2.5_ levels during walking, biking, scootering, and driving cars were 1.5–2.4 µg/m^3^ higher than the exposure during non-commuting periods. Interestingly, cleaning caused significantly less PM_2.5_ exposure in the wet season only. It is speculated that the higher relative humidity in the wet season may cause less resuspension of PM_2.5_, resulting in reduced exposure during cleaning. It should be noted that the source contribution to the PM_2.5_ exposure of these subjects was assessed previously using linear mixed-effects models [34], without removing the influence of ambient levels entirely. Using GAMM with direct deduction of concurrent ambient levels, our results showed slightly lower estimates than the previous estimates. Of course, the actual source contribution on exposure depends on the real condition. For example, in the worst situation of mosquito coil burning in this study, the 5-min peak exposure was 301.6 µg/m^3^ when the concurrent ambient level was only 46.7 µg/m^3^.

**Table 4:** Incremental contribution of different sources to 5-min PM_2.5_ and PM_1_ exposure (µg/m^3^) in (a) Indonesia and (b) Taiwan.

(a) Indonesia						
Source	Contributions (µg/m^3^)					
	All		Wet		Dry	
	PM_2.5_	PM_1_	PM_2.5_	PM_1_	PM_2.5_	PM_1_
Aromatic products	0.30	--^a^	0.50	--	--	--
Cleaning	−2.05*	--	−6.08***	--	2.19	--
Cooking	2.73***	--	3.17***	--	2.26**	--
ETS	1.86	--	−4.02	--	3.00*	--
Community factories	4.73**	--	2.72	--	7.19***	--
Mosquito coil burning	5.82***	--	8.84***	--	3.56*	--
Walking (traffic)	1.54**	--	1.19	--	1.68*	--
Biking (traffic)	2.38**	--	2.51*	--	2.05*	--
Scootering (traffic)	1.68***	--	2.14**	--	1.69**	--
Bus/Minibus (traffic)	2.21***	--	1.79	--	2.51***	--
Car (traffic)	0.52	--	1.22	--	−0.04	--
Season	2.51	--	--	--	--	--
**(b) Taiwan**						
	**All**		**Summer**		**Winter**	
Agricultural waste burning	7.17***	6.75***	0.93	0.60	17.4***	16.9***
Aromatic products	0.29	0.03	−0.76	−0.70	0.89	0.37
Cleaning	0.69	0.76	0.78	1.20	0.46	0.24
Cooking	0.54	0.49*	0.29	0.27	0.79*	0.74*
ETS	0.58	0.43	0.15	0.09	0.99	0.89
Community factories	10.1***	6.66***	12.0***	8.13***	6.36***	3.67*
Garbage burning	0.24	0.05	−0.49	−0.48	0.78	0.49
Incense burning	2.59***	1.84***	1.65*	0.9	3.53***	2.84***
Mosquito coil burning	9.82***	7.23***	-- ^b^	-- ^b^	8.32***	6.30***
Walking (traffic)	0.32	0.26	−0.09	−0.05	0.59	0.46
Biking (traffic)	0.56	0.51	0.34	0.28	0.57	0.52
Scootering (traffic)	0.33	0.27	0.26	0.22	0.40	0.35
Bus (traffic)	−0.32	−0.94	2.22	1.97	−2.88	−3.64
Car (traffic)	−1.55**	−1.44***	−1.63*	−1.53***	−1.49*	−1.31*
Season	13.7***	14.0***	--	--	--	--

***p < 0.001, **p < 0.01, *p < 0.05.

^a^No coefficients due to missing data.

^b^Omitted due to low occurrence frequency (<10).

For Taiwanese subjects, agricultural waste burning, cooking, community factories, incense burning, and mosquito coil burning were statistically significant sources for either PM_2.5_ or PM_1_ exposure when observations from both seasons were pooled (Table 4b). During commuting, only the exposure levels of car drivers were statistically significantly lower compared to those during the non-commuting periods, among all different transportation modes. Community factories, mosquito coil burning, and agricultural waste burning were the three major contributors to PM exposure. Community factories and mosquito coil burning were the sources that contributed the highest increments to subjects’ PM_2.5_ (10.1 µg/m^3^) and PM_1_ (7.23 µg/m^3^) exposure, respectively. Community factories had a higher incremental PM_2.5_ or PM_1_ contribution in summer compared to winter. In contrast, agricultural waste burning had higher incremental PM_2.5_ or PM_1_ contributions in winter compared to summer. Additionally, it is surprising to see that mosquito coil burning occurred in winter rather than in summer. Taiwanese people usually use air conditioning indoors with the windows closed during the summer, while they tend to keep their windows open in the winter. With rising temperatures due to climate change, there are more mosquitoes in winter. This may be the reason why the subjects burned mosquito coils in winter to repel mosquitoes from outdoors.

Environmental research on industrial PM emissions has focused on large industrial parks. Investigations of community air quality or residents’ exposure affected by nearby factories have been scarce. Nevertheless, due to packed living conditions in Asia and Africa, the impacts of community factories have been reported to affect personal PM exposure with traditional personal samplers [29, 30, 45]. For example, Nkhama et al. [45]. conducted a panel study near a cement factory in Zambia and found that 24-h mean PM_2.5_ concentrations ranged from 2.39 to 24.93 µg/m^3^ in the exposed community compared to 1.69–6.03 µg/m^3^ in the control community. Recently, more work has been conducted with microsensors. For example, Omokungbe et al. [46] found that daily mean PM_2.5_ level was 213.3 µg/m^3^ with low-cost sensors, compared to 20.2–44.1 µg/m^3^ in the communities further downwind of an iron smelting facility in Nigeria. In Taiwan, the subjects passing by community factories were exposed to, on average, 38.4 µg/m^3^ higher daily PM_10_ exposure than the non-exposed subjects [29]. The 5-min PM_2.5_ and PM_1_ levels near community factories in Taiwan were, on average, 6.7 ± 8.9 and 5.8 ± 8.4 µg/m^3^ higher than those at the community background location [30]. The average PM_2.5_ (10.1 µg/m^3^) and PM_1_ (6.66 µg/m^3^) exposure increment due to community factories in the current study was within the previously reported range. This also shows that community factories remain a significant PM exposure source for Taiwanese, even though the ambient PM levels have significantly reduced in the past 20 years.

Traffic emissions are the most frequent encountered source by the subjects in both countries. Microsensors have been used to assess PM exposure during commutes in several Asian countries. deSouza et al. [47]. assessed the PM_2.5_ exposure of subjects in China taking four different transportation modes, namely bike (90-min averages: 31 ± 16 µg/m^3^), bus (nearly 1-h averages: 27 ± 15 µg/m^3^), subway (100-min averages: 25 ± 13 µg/m^3^), and taxi (60–90-min averages: 20 ± 8 µg/m^3^), with Plantower PMS1003 sensors. Patra and Vanajakshi [48] have applied Sensirion SPS 30 and Panasonic PM_2.5_ microsensors to pedestrians in India at heights of 80 and 150 cm; they found that the 1-min PM_2.5_ exposure levels were 56.7 ± 6.0 and 45.6 ± 5.9 µg/m^3^ on the first day and 26.3 ± 2.4 and 21.2 ± 1.9 µg/m^3^ on the second day, respectively. Wang et al. [49] with Plantower PMS3003 microsensors, found 5-min PM_2.5_ exposure levels of subjects using six different transportation modes in Taiwan to be 17.0 ± 9.5 µg/m^3^. Wu et al. [50] found the 1-min PM_2.5_ exposure levels of bikers to be 13.5 ± 8.4, 12.9 ± 4.2, and 15.4 ± 5.4 µg/m^3^ in Taipei, Osaka, and Seoul, respectively, during rush hours with AirVisual Node. The PM exposure during commuting in this study falls within the previously reported range.

For other sources, only a few studies have assessed personal PM_2.5_ exposure with microsensors. Tsou et al. [19] assessed the PM_2.5_ and PM_1_ exposure of 35 subjects in northern Taiwan and found that the highest contribution was from incense burning, which on average contributed 9.2 µg/m^3^ for both 5-min PM_2.5_ and PM_1_ exposures, higher than the current estimates (1.8–2.6 µg/m^3^). Lung et al. [28] assessed 33 subjects’ exposures in Taiwan and found that the highest contribution was from ETS, with 8.5 µg/m^3^ increments for 30-min PM_2.5_ exposure, higher than the estimate in the current study (roughly 0.5 µg/m^3^). The distance from the smokers may be one of the reasons for this difference. In addition, Hien et al. [51] assessed visitors’ PM_2.5_ and PM_1_ exposure inside temples in Vietnam and Taiwan. They found that PM_2.5_ and PM_1_ exposure levels were 36.5 ± 33.9 and 22.7 ± 18.7 µg/m^3^ (30-min averages) in Vietnamese temples and 97.0 ± 65.4 and 74.5 ± 53.4 µg/m^3^ (40-min averages) in Taiwanese temples, respectively. The PM exposure related to incense burning in the current study is lower than their assessment. Moreover, the PM emissions from aromatic products (such as candle burning and hair spray) have been reported with traditional monitors in only a few papers [52–54], and not with sensors. The affordability and ease of use of microsensors may facilitate their use in studies on these exposure sources.

Several chamber studies have highlighted the high PM emission factors of cooking, mosquito coil burning, incense burning, and candle burning [55–58]. Liu et al. [59] also noted the prevalence of mosquito coil burning in Asia, Africa, and South America, while Yadav et al. [60] emphasized the prevalence of incense burning in eight countries, including three heavily populated Asian countries: China, India, and Indonesia. These observations further underscore the importance of studying personal PM exposure resulting from these sources, which could be assessed with microsensors as shown in this work.

### Immediate health impacts of PM_2.5_ and PM_1_ in two countries

PM levels during the non-sleeping periods used in the exposure–health evaluation are summarized in Table S2. The immediate impacts of 5-min PM_2.5_ and PM_1_ on the HRV indices and HR of the subjects are listed in Table 5. For Indonesian subjects, only LF/HF was significantly affected by PM_2.5_ and PM_1_ in the wet season (Table S3). However, when focusing on analyzing those subjects riding scooters (*n* = 13), more HRV indices were affected. Thus, Table 5a, b summarizes the results of these scooter riders. It was found that more HRV indices reached statistically significant impacts associated with PM_1_ than PM_2.5_, and in the dry season than the wet season. In the dry season, on average, a −3.1% to −5.7% change in SDNN, LF/HF, LF, VLF, and TP were observed for a 10 µg/m^3^ increase in PM_2.5_. For a 10 µg/m^3^ increase in PM_1_, on average, a −2.5% to −6.7% change in SDNN, RMSSD, LF/HF, LF, VLF, and TP were observed in the dry season. Just for reference, the interquartile ranges (IQR) for the PM_2.5_ and PM_1_ exposure levels of Indonesian subjects during non-sleeping periods were 17.3 and 15.4 µg/m^3^, respectively. Additionally, the percentage changes of these significant HRV indices were all consistently higher when associated with PM_1_ than PM_2.5_. Additionally, no statistically significant lag effects on HRV indices were observed for these subjects (Fig. S4).

**Table 5:** Impacts of 5-min PM exposure on the HRV indices and HR of the subjects. (a) PM_2.5_ and (b) PM_1_ impacts for the Indonesia scooter group (*n* = 13) and (c) PM_2.5_ and (d) PM_1_ impacts for all Taiwanese subjects (*n* = 51). Numbers presented are changes for a 10 μg/m^3^ increase in PM; 95% confidence intervals are listed in the parentheses.

(a) Indonesia PM_2.5_								
	SDNN	RMSSD	LF/HF	HF	LF	VLF	TP	HR
All								
PM_2.5_	−0.4 (−1.0, 0.2)	−0.5 (−1.2, 0.3)	1.0* (0.1, 2.0)	−1.2 (−2.7, 0.2)	−0.7 (−1.8, 0.5)	−0.6 (−1.9, 0.6)	−0.6 (−1.7, 0.5)	0.0 (−0.1, 0.2)
Outdoor	−18.4*** (−21.9, −14.8)	−12.3*** (−16.8, −7.6)	4.0 (−2.7, 11.2)	−27.3*** (−34.4, −19.5)	−27.9*** (−33.7, −21.7)	−34.9*** (−40.4, −28.9)	−31.1*** (−36.4, −25.3)	−0.3 (−1.3, 0.7)
Season	13.0*** (5.4, 21.2)	39.6*** (25.5, 55.3)	−22.4*** (−30.3, −13.7)	93.5*** (60.3, 133.7)	52.5*** (−33.7, −21.7)	10.1 (−3.8, 26.0)	27.4*** (−36.4, −25.3)	2.9 (−0.5, 6.4)
Wet								
PM_2.5_	−0.2 (−0.8, 0.4)	−0.3 (−1.0, 0.5)	1.2* (0.2, 2.3)	−1.1 (−2.5, 0.4)	−0.4 (−1.5, 0.7)	−0.1 (−1.4, 1.2)	−0.3 (−1.4, 0.9)	0.1 (−0.1, 0.2)
Outdoor	−19.9*** (−24.0, −15.6)	−15.0*** (−20.3, −9.5)	15.3** (5.9, 25.6)	−32.2*** (−40.0, −23.3)	−28.3*** (−34.8, −21.2)	−35.7*** (−42.3, −28.3)	−33.0*** (−39.1, −26.3)	0.7 (−0.5, 1.9)
Dry								
PM_2.5_	−3.1** (−4.9, −1.2)	−1.9 (−4.0, 0.3)	−3.1** (−5.3, −0.8)	−1.8 (−5.7, 2.3)	−5.7** (−9.1, −2.1)	−5.6** (−9.0, −2.1)	−4.9** (−8.1, −1.6)	−0.3 (−0.8, 0.2)
Outdoor	−15.7*** (−22.3, −8.7)	−7.7 (−15.7, 1.1)	−9.6* (−18.0, −0.3)	−19.0* (−32.0, −3.5)	−23.4*** (−34.5, −10.5)	−32.9*** (−42.6, −21.6)	−26.3*** (−36.4, −14.6)	−1.7 (−3.4, 0.0)
(b) Indonesia PM_1_								
All								
PM_1_	−0.9* (−1.7, −0.1)	−1.2* (−2.3, −0.1)	1.4* (0.0, 2.7)	−2.4* (−4.4, −0.3)	−1.5 (−3.1, 0.2)	−1.5 (−3.2, 0.1)	−1.4 (−2.9, 0.2)	−0.2 (−0.4, 0.1)
Outdoor	−18.3*** (−21.8, −14.6)	−12.1*** (−16.6, −7.3)	3.8 (−2.9, 11.0)	−27.0*** (−34.1, −19.2)	−27.7*** (−33.5, −21.5)	−34.7*** (−40.2, −28.6)	−30.9*** (−36.3, −25.1)	−0.3 (−1.3, 0.7)
Season	13.5*** (5.8, 21.7)	40.6*** (26.4, 56.4)	−22.6*** (−30.5, −13.9)	95.4*** (61.8, 136.1)	53.7*** (33.3, 77.3)	10.9 (−3.1, 27.1)	28.3*** (12.9, 45.9)	3.2 (−0.3, 6.7)
Wet								
PM_1_	−0.5 (−1.4, 0.4)	−1.0 (−2.2, 0.3)	1.9* (0.3, 3.5)	−2.4* (−4.7, −0.0)	−0.9 (−2.5, 0.8)	−0.7 (−2.5, 1.2)	−0.8 (−2.4, 0.8)	−0.1 (−0.4, 0.2)
Outdoor	−19.8*** (−24.0, −15.5)	−14.8*** (−20.1, −9.3)	14.9** (5.5, 25.2)	−31.9*** (−39.8, −23.0)	−28.2*** (−34.7, −21.1)	−35.5*** (−42.2, −28.1)	−32.9*** (−39.0, −26.1)	0.7 (−0.5, 1.9)
Dry								
PM_1_	−3.8*** (−5.8, −1.7)	−2.5* (−4.9, −0.0)	−3.4* (−5.9, −0.8)	−2.6 (−7.0, 2.1)	−6.7** (−10.6, −2.7)	−6.7** (−10.5, −2.7)	−6.0** (−9.6, −2.2)	−0.6 (−1.1, 0.0)
Outdoor	−15.5*** (−22.0, −8.4)	−7.4 (−15.5, 1.4)	−9.4* (−17.9, −0.1)	−18.6* (−31.7, −3.0)	−23.0** (−34.2, −10.0)	−32.6*** (−42.3, −21.2)	−25.9*** (−26.0, −14.4)	−1.7 (−3.4, 0.1)
(c) Taiwan PM_2.5_								
All								
PM_2.5_	−1.0*** (−1.6, −0.5)	−0.6 (−1.2, 0.1)	1.0* (0.0, 2.0)	−1.8** (−3.2, −0.4)	−1.1 (−2.2, 0.1)	−1.5* (−2.6, −0.4)	−1.5** (−2.5, −0.4)	0.3*** (0.2, 0.4)
Outdoor	−5.0*** (−6.8, −3.2)	−2.0 (−4.1, 0.1)	0.1 (−3.0, 3.3)	−3.8 (−8.1, 0.8)	−4.3* (−8.0, −0.5)	−9.5*** (−12.8, −6.1)	−7.8*** (−10.9, −4.5)	0.7*** (0.3, 1.0)
Season	−6.2*** (−7.8, −4.5)	−13.1*** (−15.2, −11.1)	8.1*** (4.8, 11.4)	−17.3*** (−8.1, −0.8)	−10.4*** (−13.7, −6.8)	−7.3*** (−10.5, −4.0)	−9.6*** (−12.6, −6.5)	4.1*** (3.2, 5.0)
Summer								
PM_2.5_	−1.8*** (−2.7, −1.0)	−1.3** (−2.2, −0.3)	1.6* (0.1, 3.1)	−4.0*** (−5.9, −2.0)	−2.8** (−4.5, −1.0)	−2.6** (−4.3, −0.9)	−2.7*** (−4.3, −1.1)	0.4*** (0.2, 0.5)
Outdoor	−7.6*** (−10.2, −4.9)	−1.1 (−4.3, 2.3)	−3.2 (−7.8, 1.7)	−1.1 (−7.8, 6.1)	−4.5 (−10.1, 1.4)	−14.3*** (−19.1, −9.3)	−10.8*** (−15.5, −5.9)	1.0** (0.4, 1.6)
Winter								
PM_2.5_	−0.5 (−1.3, 0.3)	0.0 (−0.8, 0.9)	0.5 (−0.9, 1.8)	−0.1 (−2.0, 1.8)	0.3 (−1.3, 1.9)	−1.0 (−2.6, 0.6)	−0.6 (−2.1, 0.9)	0.3*** (0.1, 0.4)
Outdoor	−3.9*** (−6.2, −1.6)	−3.6** (−6.3, −0.9)	2.4 (−1.7, 6.7)	−7.2* (−12.6, −1.6)	−5.9* (−10.5, −1.1)	−7.3** (−11.6, −2.7)	−7.1** (−11.2, −2.7)	0.5* (0.0, 1.0)
(d) Taiwan PM_1_								
All								
PM_1_	−1.2*** (−2.0, −0.5)	−1.0* (−1.8, −0.2)	1.6* (0.3, 2.9)	−2.5** (−4.3, −0.7)	−1.1 (−2.7, 0.4)	−1.7* (−3.2, −0.3)	−1.8* (−3.1, −0.4)	0.5*** (0.3, 0.6)
Outdoor	−5.0*** (−6.8, −3.2)	−2.0 (−4.1, 0.1)	0.1 (−3.0, 3.3)	−3.7 (−8.1, 0.8)	−4.3* (−8.0, −0.5)	−9.5*** (−12.8, −6.1)	−7.7*** (−10.9, −4.4)	0.7*** (0.3, 1.0)
Season	−6.0*** (−7.7, −4.3)	−12.8*** (−14.9, −10.6)	7.4*** (4.1, 10.9)	−16.8*** (−20.7, −12.7)	−10.4*** (−13.9, −6.7)	−7.2*** (−10.5, −3.8)	−9.4*** (−12.5, −6.2)	3.9*** (3.0, 4.8)
Summer								
PM_1_	−2.4*** (−3.8, −1.0)	−2.1** (−3.6, −0.6)	4.0** (1.6, 6.5)	−6.4*** (−9.5, −3.2)	−3.1* (−5.9, −0.3)	−3.1* (−5.8, −0.3)	−3.4* (−5.9, −0.8)	0.6*** (0.4, 0.9)
Outdoor	−7.6*** (−10.2, −4.9)	−1.0 (−4.3, 2.3)	−3.3 (−7.9, 1.6)	−1.0 (−7.7, 6.2)	−4.4 (−10.0, 1.5)	−14.3*** (−19.1, −9.2)	−10.7*** (−15.4, −5.8)	1.0** (0.4, 1.6)
Winter								
PM_1_	−0.8 (−1.6, 0.1)	−0.3 (−1.4, 0.7)	0.6 (−0.9, 2.2)	−0.7 (−2.8, 1.5)	−0.1 (−1.9, 1.8)	−1.4 (−3.2, 0.3)	−1.1 (−2.7, 0.6)	0.4*** (0.2, 0.6)
Outdoor	−3.9** (−6.2, −1.6)	−3.7** (−6.3, −0.9)	2.4 (−1.7, 6.7)	−7.3* (−12.6, −1.6)	−5.9* (−10.5, −1.1)	−7.3** (−11.7, −2.7)	−7.1** (−11.2, −2.7)	0.5* (0.0, 1.0)

*** *p* < 0.001, ** *p* < 0.01, * *p* < 0.05.

For Taiwanese subjects, HR and most HRV indices showed statistically significant impacts associated with both PM_2.5_ and PM_1_, with more statistically significant impacts in summer than in winter (Table 5c, d). In summer, a −1.3% to −4.0% change was observed for SDNN, RMSSD, HF, LF, VLF, and TP, for a 10 µg/m^3^ increase in PM_2.5_, and a −2.1% to −6.4% change, for a 10 µg/m^3^ increase in PM_1._ For reference, the IQRs for PM_2.5_ and PM_1_ exposure of Taiwanese subjects during non-sleeping periods were 12.9 and 12.4 µg/m^3^, respectively. Again, the percentage changes of these HRV indices and HR are consistently higher when associated with PM_1_ than for PM_2.5_; and no statistically significant lag effects were observed (Fig. S5).

Comparing the HRV changes in both countries, more HRV indices with statistically significant were observed in Taiwanese subjects compared to Indonesian scooter riders; this may due to much larger sample size in Taiwan (*n* = 33,125) than in Indonesia (*n* = 5946). When comparing the impacts of the dry season in Indonesia with those in the summer in Taiwan, it was found that the majority of percentage changes in significant HRV indices were slightly higher in Indonesia than in Taiwan. Age was already adjusted in the exposure–health models, but the inherent difference in the age distribution of these two groups may be the reason for these differences. Another interesting point is that the outdoor environment is a significant factor in the relationship between PM and HRV for both countries, with higher impacts observed in Indonesia. These models were re-run without considering the “outdoor” factor, and the results showed that most of the coefficients of PM changed only slightly (Tables S4, S5), indicating that the impact of the outdoor environment was associated with other factors. Activity level, which is one potential factor that may differ between indoor and outdoor microenvironments, was adjusted; thus, the underlying reasons for this significant outdoor impact on HRV require further investigation.

A meta-analysis including 26 epidemiological studies with 24-h or 48-h exposure reported that, on average, a −1.25% to −3.17% change in SDNN, RMSSD, LF, and HF were observed for a 10 µg/m^3^ increase in PM_2.5_ [61]. A recent meta-analysis focusing on older adults with 19 longitudinal studies found that for short-term exposure (days or weeks), a 10 µg/m^3^ increase in PM_2.5_ was associated with a −0.39% to −2.31% change in SDNN, RMSSD, LF, and HF [62]. The HRV changes found in this study fall within the aforementioned range. Additionally, in Taiwan, Huang et al. [39] assessed PM_2.5_ impacts on HRV for 50 housewives and found that a 1-h mean PM_2.5_ was associated with a −1.25% change in SDNN for an IQR change (19.8 µg/m^3^) in household PM_2.5_ exposure; the HRV change was twice the magnitude of our estimates, possibly due to younger subjects with a mean age of 38 ± 10.5 years in their study.

### Overall discussion and implication

Most PM and health studies have typically focused on assessing the short-term (days or weeks) or long-term effects (months or longer) of PM exposure based on daily health records and hourly (or daily) exposure. This study stands out as one of the few to demonstrate the immediate health impacts of peak PM in the resolution of minutes, taking advantage of lightweight, low-cost, and portable microsensors in both the environmental and health fields. It was found that both PM_2.5_ and PM_1_ have immediate impacts on HRV indices, which are linked to an increased risk of heart attack. The technology and methodology employed in this study offer great potential for researchers in the resource-limited countries with high levels of PM_2.5_ and PM_1_. By utilizing these tools, it becomes possible to identify the sources responsible for peak PM exposure and implement targeted interventions to reduce immediate health impacts, whether through source control measures or behavior change strategies.

Our results show that community factories remain important exposure sources in Taiwan over the past 20 years, even though the ambient air quality, measured by monitoring stations situated at 10-m above the ground, has improved significantly. Community factories have been largely unsupervised, as most control strategies have focused on large industrial plants and traffic emissions. Lessons learned from Taiwan are applicable to other Asian countries with similar cultures and living styles. Many of them emphasize economic development with inadequate zoning regulations resulting in factories without proper controls scattered throughout communities. It is crucial to devise adequate zoning regulations to eliminate community factories and enforce proper control strategies for mid- or small-scale factories to reduce residents’ exposure to community factory emissions. To ensure that authorities implement appropriate measures, scientists must play an active role in providing solid evidence. The availability of low-cost microsensors and the methodology presented in this work can serve as examples to facilitate scientists in limited-funding Asian countries in conducting PM exposure assessment, source identification/quantification, and health evaluation. Implementing proper controls for the identified PM sources within communities can lower peak PM exposures and reduce the likelihood of certain lethal health impacts such as stroke.

Our findings highlight the importance of implementing targeted control measures for nearby sources, which may not currently be the primary focus of pollution mitigation efforts. By introducing measures to reduce emissions from these sources, significant reductions in peak exposure and associated immediate health impacts can be achieved. Furthermore, promoting behavior changes that address personal activities contributing to PM exposure, such as reducing the burning of scented candles (aromatic products), agricultural waste, incense, and mosquito coils, can further contribute to lowering individuals’ PM exposure levels. Overall, the application of microsensors, combined with targeted source control and behavior change interventions, presents substantial potential for reducing PM-related health risks, improving air quality in Asian countries, and thus creating a healthier environment for the population.

Based on the assessment from multiple angles, it was found that community factories and mosquito coil burning are two prominent exposure sources that contribute significantly to subjects’ exposure in both countries. Surprisingly, their contributions were even greater than those of ETS and traffic emissions, which are typically emphasized in the literature. The two sources were associated with the elevated levels of PM_2.5_ and PM_1_ peaks, accounting for a substantial proportion of the 24-h exposure, and contributed statistically significant increments compared to non-exposed periods. Given these findings, prioritizing interventions to mitigate the impacts of these sources is crucial for public health protection. While measures targeting industrial parks and motor vehicles remain important, focusing on these identified sources will provide an additional layer of protection. By effectively addressing these significant exposure sources, public health can be better safeguarded against the adverse effects of PM_2.5_ and PM_1_ pollution.

In terms of immediate health impacts, it was found that the impacts in the dry season and summer for Indonesian and Taiwanese subjects, respectively, were more severe than those in the wet season and winter, respectively. For scooter riders in Indonesia, a −3.1% to −5.7% and a −2.5% to −6.7% change in HRV indices were observed for a 10 µg/m^3^ increase in PM_2.5_ and PM_1_, respectively, in the dry season. For all Taiwanese subjects, the corresponding changes were −1.3% to −4.0% and −2.1% to −6.4%, respectively, in the summer, except for LF/HF. These findings align with previous studies conducted mostly in developed countries. Moreover, the immediate health impacts of PM_1_ were more severe than those of PM_2.5_, again emphasizing the urgent need to assess exposure and health impacts of PM_1_. Microsensors are valuable and affordable tools for low- and middle-income countries to evaluate the exposure and health impacts of PM_2.5_ and PM_1_, especially during their developing stages when pollution levels tend to be higher. This work serves as a good demonstration.

The comparison between the two panels is summarized here. Indonesian subjects were generally exposed to higher PM_2.5_ and PM_1_ levels than those in Taiwan; nevertheless, the PM_1_/PM_2.5_ ratios of the subjects’ exposure were similar (around 0.9) in both countries. Even though traffic emissions were the most frequently observed exposure sources, community factories and mosquito coil burning were the two most important exposure sources in both countries. These sources were associated with the highest medians and 95th percentiles of PM_2.5_ levels among all sources and the highest average incremental contribution to PM exposure after adjusting for concurrent ambient levels. Moreover, the HRV indices were affected by 5-min peak PM_2.5_ and PM_1_ exposure for subjects in both countries, with seasonal variations. The group of scooter riders in Indonesia had more HRV indices with statistically significant changes compared to the overall group. When comparing the HRV changes of scooter riders in the dry season of Indonesia with those of all subjects in summer in Taiwan, it was found that most percentage changes were higher in Indonesia than in Taiwan, possibly due to the younger age group in Indonesia. Furthermore, the impacts of HRV changes associated with PM_1_ were greater than those associated with PM_2.5_ in both countries.

Wearable sensors have been applied to assess the vital signs of subjects in various studies; however, most of the wearable sensors used in the literature were not medically certified [63–65]. Thus, the validity of the measurements is in question. In terms of heart signals, only HR is available, but not HRV indices in those consumer products. Assessing HRV indices is important in studying the impacts of peak PM exposure, since changes in these indices are associated with an increased risk of heart attack [21]. This work used a medically-certified sensor for HRV indices along with PM sensors, providing valuable evidence of the immediate health impacts of peak PM_2.5_ and PM_1_ exposure in two Asian countries.

Our study has several limitations. To match the 5-min resolution of HRV indices in health evaluation, the 15-s resolution of PM peaks and the 30-min resolution of TAD records were re-processed as 5-min. Thus, the peak values reported are underestimates. Moreover, it was assumed that all 5-min segments in a 30-min TAD record had the same exposure sources, which may result in underestimated source contributions in the GAMM analysis. Furthermore, only the primary exposure sources were used in the analysis, so the exposure frequency may be underestimated for certain sources. Nevertheless, the above limitations result in underestimates rather than overestimates of the source contribution and health impacts. Thus, these limitations do not affect the validity of our results. Additionally, our findings may have been confounded by other unmeasured air pollutants. Further investigation is needed to better understand the potential confounding effects of these pollutants. Besides, the number of subjects was limited in both countries; therefore, the generalization of the results is also limited to groups with similar characteristics. The occupational distributions of the subjects did not necessarily represent the occupational distribution of the general public in either country. However, the subjects did encompass a range of occupations, including white-collar workers, blue-collar workers, and housewives/unemployed individuals. Consequently, our findings, such as the identified exposure sources, were not biased toward any particular occupation. Overall, while acknowledging these limitations, our study still provides valuable insights into the assessment of exposure sources and the immediate health impacts of PM_2.5_ and PM_1_.

This study applied calibrated PM microsensors and medically-certified HRV microsensors to assess peak exposure, exposure sources, and immediate HRV changes due to PM_2.5_ and PM_1_ exposure in Indonesia and Taiwan. It demonstrates the advantages of utilizing high-resolution microsensors in PM exposure assessment and health evaluation. These microsensors serve as valuable tools for scientists in resource-limited countries where PM pollution is severe and targeted control measures are urgently needed. The identified important exposure sources serve as a primer for scientists in other countries with similar cultures and living styles to investigate their own exposure sources. The methodology could be applied to these countries to identify sources responsible for peak PM exposure. These findings can prompt authorities to revise zoning and control regulations, ultimately leading to a much healthier community environment.

## Supplementary information

Supplementary Information

## Data Availability

The environmental datasets generated during and/or analyzed during the current study are available in the [depositar] repository. Available from: https://data.depositar.io/dataset/pm_data_taiwan_indonesia. The health datasets generated in the current study are not publicly available due to privacy consideration; the summary tables are shown in the paper.
