# Supplementary material for: Peaks, sources, and immediate health impacts of PM2.5 and PM1 exposure in Indonesia and Taiwan with microsensors
Source: J Expo Sci Environ Epidemiol. 2024 May 28;35(2):264–77. doi: 10.1038/s41370-024-00689-4 (PMC12009734; doi:10.1038/s41370-024-00689-4)
Supplement: Supplementary file 1 — Supplementary Information [file 41370_2024_689_MOESM1_ESM.pdf]

Supplementary Information

Peaks, Sources, and Immediate Health Impacts of $PM_{2.5}$ and PM_1 Exposure in Indonesia and Taiwan with Microsensors

Shih-Chun Candice Lung[a,b,c*], Ming-Chien Mark Tsou[a], Chih-Hui Chloe Cheng[a], and Wiwiek Setyawati[d]

[a]Research Center for Environmental Changes, Academia Sinica, Taipei, Taiwan.

[b]Department of Atmospheric Sciences, National Taiwan University, Taipei, Taiwan.

[c]Institute of Environmental and Occupational Health Sciences, National Taiwan University, Taipei, Taiwan.

[d]Research Center for Climate and Atmosphere, National Research and Innovation Agency (BRIN), Kota Bandung, Indonesia.

[*]Corresponding author, Research Center for Environmental Changes, Academia Sinica, No. 128, Sec. 2, Academia Rd, Nangang, Taipei, Taiwan 11529; Tel: 886-2-27875908, Fax: 886-2-27833584, email: *sclung@rcec.sinica.edu.tw.*

This Supplementary Information file contains five tables and five figures presenting more details of the exposure levels and the statistical results of the exposure-health relationships for $PM_{2.5}$ and PM_1. The titles of these tables and figures ae listed in the next page.

Table S1 Summary of various exposure sources specified in the time-activity diaries in (a) Indonesia and (b) Taiwan, regarding the maximum 5-min $PM_{2.5}$ peak of that source, the ratio of that peak over the mean value of that hour, and the exposure percentage of that source accounted for the 24-hour exposure.

Table S2 Summary of personal PM exposure levels in (a) Indonesia and (b) Taiwan during the non-sleeping periods used in the exposure-health evaluation.

Table S3 Impacts of 5-min (a) $PM_{2.5}$ and (b) PM_1 exposures on the heart rate variability (HRV) indices and heart rate (HR) of Indonesian subjects (n=49).

Table S4 Impacts of 5-min PM exposures on the HRV indices and HR of the subjects. (a) $PM_{2.5}$ and (b) PM_1 impacts for the Indonesian scooter group (n=13) and (c) $PM_{2.5}$ and (d) PM_1 impacts for all Taiwanese subjects (n=51) after removing "outdoor" factor from the models.

Table S5 Impacts of 5-min (a) $PM_{2.5}$ and (b) PM_1 exposures on the heart rate variability (HRV) indices and heart rate (HR) of Indonesian subjects (n=49) after removing "outdoor" factor from the models.

Figure S1 Examples of $PM_{2.5}$ exposures during commute with scooters in (a) Indonesia and (b) Taiwan.

Figure S2 Frequency distribution of various exposure sources or commuting modes (in 5-min resolution) in (a) Indonesia and (b) Taiwan.

Figure S3 The distribution of 5-min $PM_1/PM_{2.5}$ ratios while exposing to different sources or during different commuting modes in (a) Indonesia and (b) Taiwan.

Figure S4 Lag effects of 5-min (a) $PM_{2.5}$ and (b) PM_1 exposures on the HRV indices and HR of the Indonesia scooter group (n=13). Numbers presented are changes for a 10 $\mu g/m^3$ increase in PM.

Figure S5 Lag effects of 5-min (a) $PM_{2.5}$ and (b) PM_1 exposures on the HRV indices and HR of all Taiwanese subjects (n=51). Numbers presented are changes for a 10 $\mu g/m^3$ increase in PM.

Table S1 Summary of various exposure sources specified in the time-activity diaries in (a) Indonesia and (b) Taiwan, regarding the maximum 5-min $PM_{2.5}$ peak of that source, the ratio of that peak over the mean value of that hour, and the exposure percentage of that source accounted for the 24-hour exposure (summation of $PM_{2.5}$ exposure of that source in that day over the total 24-hour $PM_{2.5}$ exposure). The top two in each country are shown in bold.

(a) Indonesia

**Source**	**maximum 5-min $PM_{2.5}$ peak**	**maximum/hourly-mean ratio**	**% of that source in 24-hour period**
Aromatic products	94.3	1.6	**42.0%**
Cleaning	147.1	2.3	4.0%
Cooking	**473.6**	**4.9**	7.0%
ETS	161.2	2.8	16.1%
Community factory	163.0	1.2	15.0%
Mosquito coil burning	**301.6**	**3.5**	**18.0%**
Walking (traffic)	102.3	1.5	16.5%
Biking (traffic)	208.7	1.7	16.4%
Scootering (traffic)	106.5	2.2	3.6%
Bus (traffic)	99.8	1.9	9.4%
Car (traffic)	106.4	1.5	9.1%

(b) Taiwan

**Source**	**maximum five-min $PM_{2.5}$ peak**	**maximum/hourly-mean ratio**	**% of that source in 24-hour period**
Agriculture burning	105.8	2.1	5.6%
Aromatic products	22.7	1.0	4.1%
Cleaning	88.0	1.5	9.5%
Cooking	88.6	2.5	24.1%
ETS	148.1	1.8	**39.8%**
Community factory	**467.4**	2.4	**37.1%**
Garbage burning	39.5	1.1	2.8%
Incense burning	171.0	**3.4**	7.3%
Mosquito coil burning	**361.3**	2.2	21.2%
Walking (traffic)	102.4	1.7	5.1%
Biking (traffic)	35.8	1.2	4.0%
Scootering (traffic)	112.4	**3.6**	10.3%
Bus (traffic)	75.6	3.1	11.5%
Car (traffic)	84.0	2.7	11.3%

Table S2 Summary of personal PM exposure levels in (a) Indonesia and (b) Taiwan during the non-sleeping periods used in the exposure-health evaluation.

	Season	*n*	$PM_{2.5}$ (µg/m^3)		PM_1 (µg/m^3)	
			**Mean (SD)**	**Max**	**Mean (SD)**	**Max**
**(a) Indonesia**						
	All	21519	29.1 (17.6)	473.6	25.9 (14.0)	154.0
	Wet	12489	28.3 (19.6)	473.6	25.2 (15.3)	152.7
	Dry	9030	30.1 (14.3)	301.6	26.8 (11.9)	154.0
**(b) Taiwan**						
	All	33125	15.9 (12.2)	467.4	14.8 (10.4)	217.7
	Summer	15782	9.5 (10.2)	467.4	8.7 (6.5)	120.9
	Winter	17343	21.6 (10.9)	361.3	20.3 (10.1)	217.7

Table S3 Impacts of 5-min (a) $PM_{2.5}$ and (b) PM_1 exposures on the heart rate variability (HRV) indices and heart rate (HR) of Indonesian subjects (n=49). Numbers presented are changes for a 10 μg/m^3 increase in PM; 95% confidence intervals are listed in the parentheses.

(a) Indonesia $PM_{2.5}$

	SDNN	RMSSD	LF/HF	HF	LF	VLF	TP	HR
**All**								
**$PM_{2.5}$**	0.1<br>(-0.4, 0.5)	-0.2<br>(-0.7, 0.4)	0.9 *<br>(0.1, 1.6)	-0.5<br>(-1.6, 0.6)	0.2<br>(-0.8, 1.1)	0.3<br>(-0.7, 1.3)	0.3<br>(-0.6, 1.2)	0.0<br>(-0.1, 0.1)
**Outdoor**	-11.9 ***<br>(-14.1, -9.6)	-10.4 ***<br>(-12.9, -7.8)	-1.2<br>(-5.1, 2.9)	-21.4 ***<br>(-25.9, -16.6)	-23.1 ***<br>(-27.0, -19.1)	-20.3 ***<br>(-24.3, -16.0)	-19.7 ***<br>(-23.4, -15.9)	2.7 ***<br>(2.1, 3.4)
**Season**	-9.0 ***<br>(-11.7, -6.2)	-8.7 ***<br>(-12.6, -4.7)	3.9<br>(-1.0, 2.9)	-12.4 **<br>(-19.2, -5.1)	-9.2 **<br>(-14.9, -3.1)	-15.1 ***<br>(-20.0, -10.0)	-13.5 ***<br>(-18.2, -8.6)	6.9 ***<br>(5.4, 8.5)
**Wet**								
**$PM_{2.5}$**	-0.1<br>(-0.6, 0.4)	-0.0<br>(-0.7, 0.6)	1.0 *<br>(0.1, 1.9)	-0.5<br>(-1.7, 0.8)	-0.1<br>(-1.1, 1.0)	-0.0<br>(-1.1, 1.1)	-0.1<br>(-1.1, 0.9)	0.0<br>(-0.1, 0.2)
**Outdoor**	-12.2 ***<br>(-15.0, -9.2)	-12.8 ***<br>(-16.1, -9.4)	2.6<br>(-3.0, 8.6)	-24.1 ***<br>(-29.9, -17.7)	-23.2 ***<br>(-28.2, -17.8)	-20.5 ***<br>(-25.8, -14.9)	-20.0 ***<br>(-24.8, -14.9)	2.8 ***<br>(1.9, 3.7)
**Dry**								
**$PM_{2.5}$**	-0.3<br>(-1.3, 0.6)	-0.8<br>(-1.9, 0.2)	-0.1<br>(-1.4, 1.3)	-0.9<br>(-3.0, 1.2)	-0.7<br>(-2.5, 1.1)	-0.3<br>(-2.2, 1.6)	-0.2<br>(-1.9, 1.5)	-0.0<br>(-0.2, 0.2)
**Outdoor**	-12.2 ***<br>(-15.5, -8.8)	-9.3 ***<br>(-13.0, -5.4)	-1.7<br>(-7.1, 4.0)	-21.7 ***<br>(-28.2, -14.6)	-23.5 ***<br>(-29.2, -17.4)	-20.7 ***<br>(-26.7, -14.2)	-20.4 ***<br>(-25.8, -14.5)	2.8 ***<br>(1.9, 3.6)

(b) Indonesia PM_1

	SDNN	RMSSD	LF/HF	HF	LF	VLF	TP	HR
**All**								
**PM_1**	-0.0	-0.5	1.0 *	-0.8	-0.0	0.1	0.2	-0.1
	(-0.6, 0.6)	(-1.2, 0.3)	(0.0, 2.0)	(-2.3, 0.7)	(-1.3, 1.3)	(-1.1, 1.4)	(-0.9, 1.4)	(-0.3, 0.1)
**Outdoor**	-11.9 ***	-10.4 ***	-1.2	-21.3 ***	-23.1 ***	-20.2 ***	-19.7 ***	2.8 ***
	(-14.1, -6.1)	(-12.4, -4.5)	(-5.1, 2.8)	(-25.9, -16.5)	(-26.9, -19.0)	(-24.3, -16.0)	(-23.4, -15.8)	(2.1, 3.4)
**Season**	-8.9 ***	-8.5 ***	3.9	-12.2 **	-9.0 **	-15.0 ***	-13.4 ***	7.0 ***
	(-11.7, -6.1)	(-12.4, -4.5)	(-1.0, 9.1)	(-19.0, -4.9)	(-14.8, -2.9)	(-19.9, -9.9)	(-18.1, -8.4)	(5.5, 8.6)
**Wet**								
**PM_1**	-0.2	-0.4	1.3 *	-1.0	-0.3	-0.4	-0.4	-0.1
	(-1.0, 0.5)	(-1.3, 0.5)	(0.1, 2.6)	(-2.8, 0.9)	(-1.7, 1.2)	(-1.8, 1.1)	(-1.7, 1.0)	(-0.3, 0.1)
**Outdoor**	-12.1 ***	-12.7 ***	2.5	-23.9 ***	-23.1 ***	-20.4 ***	-19.9 ***	2.8 ***
	(-15.0, -9.1)	(-16.0, -9.3)	(-3.1, 8.5)	(-29.8, -17.6)	(-28.2, -17.7)	(-25.7, -14.7)	(-24.7, -14.8)	(2.0, 3.7)
**Dry**								
**PM_1**	-0.7	-1.2	-0.4	-1.5	-1.6	-0.7	-0.7	-0.1
	(-1.8, 0.5)	(-2.4, 0.1)	(-2.1, 1.2)	(-4.0, 1.1)	(-3.8, 0.7)	(-3.0, 1.6)	(-2.9, 1.4)	(-0.4, 0.2)
**Outdoor**	-12.2 ***	-9.2 ***	-1.6	-21.6 ***	-23.4 ***	-20.6 ***	-20.3 ***	2.8 ***
	(-15.5, -8.7)	(-13.0, -5.4)	(-7.0, 4.0)	(-28.1, -14.5)	(-29.0, -17.3)	(-26.6, -14.1)	(-25.8, -14.4)	(1.9, 3.6)

Note: ***: $p < 0.001$, **: $p < 0.01$, *: $p < 0.05$.

Table S4 Impacts of 5-min PM exposures on the HRV indices and HR of the subjects. (a) $PM_{2.5}$ and (b) PM_1 impacts for the Indonesian scooter group (n=13) and (c) $PM_{2.5}$ and (d) PM_1 impacts for all Taiwanese subjects (n=51) after removing "outdoor" factor from the models. Numbers presented are changes for a 10 μg/m^3 increase in PM; 95% confidence intervals are listed in the parentheses. Bold numbers indicate that the statistically significant levels have changed compared to the results from the original model with "outdoor".

(a) Indonesia $PM_{2.5}$

	SDNN	RMSSD	LF/HF	HF	LF	VLF	TP	HR
**All**								
**$PM_{2.5}$**	-0.5<br>(-1.2, 0.1)	-0.5<br>(-1.2, 0.3)	1.0 *<br>(0.1, 2.0)	-1.4<br>(-2.8, 0.1)	-0.8<br>(-2.0, 0.4)	-0.8<br>(-2.1, 0.4)	-0.7<br>(-1.9, 0.4)	0.0<br>(-0.1, 0.2)
**Season**	8.4 *<br>(0.8, 16.5)	36.2 ***<br>(22.2, 51.8)	-21.8 ***<br>(-29.6, -13.1)	85.0 ***<br>(52.8, 123.9)	44.3 ***<br>(24.5, 67.2)	1.3<br>(-11.9, 16.4)	18.2 *<br>(3.7, 34.8)	2.8<br>(-0.5, 6.3)
**Wet**								
**$PM_{2.5}$**	-0.3<br>(-0.9, 0.4)	-0.3<br>(-1.1, 0.5)	1.3 *<br>(0.3, 2.3)	-1.2<br>(-2.7, 0.3)	-0.4<br>(-1.6, 0.7)	-0.2<br>(-1.6, 1.1)	-0.4<br>(-1.5, 0.8)	0.1<br>(-0.1, 0.2)
**Dry**								
**$PM_{2.5}$**	-3.5 ***<br>(-5.4, -1.7)	-2.0<br>(-4.2, 0.1)	-3.4 **<br>(-5.6, -1.1)	-2.3<br>(-6.2, 1.8)	**-6.3** ***<br>**(-9.7, -2.7)**	**-6.8** ***<br>**(-10.2, -3.2)**	**-5.7** ***<br>**(-9.0, -2.4)**	-0.3<br>(-0.8, 0.2)

(b) Indonesia PM_1

	SDNN	RMSSD	LF/HF	HF	LF	VLF	TP	HR
**All**								
**PM_1**	**-1.2** **	-1.4 *	1.4 *	-2.7 *	**-1.9** *	**-2.0** *	**-1.8** *	-0.2
	**(-2.0, -0.3)**	(-2.5, -0.2)	(0.1, 2.8)	(-4.8, -0.6)	**(-3.5, -0.2)**	**(-3.7, -0.3)**	**(-3.4, -0.2)**	(-0.4, 0.1)
**Season**	9.0 *	37.4 ***	-22.0 ***	87.5 ***	45.8 ***	2.3	19.5 **	3.1
	(1.4, 17.3)	(23.2, 53.1)	(-29.9, -13.3)	(54.8, 127.1)	(25.8, 69.1)	(-11.0, 17.7)	(4.8, 36.2)	(-0.3, 6.6)
**Wet**								
**PM_1**	-0.7	-1.2	**2.1** **	-2.8 *	-1.1	-1.1	-1.2	-0.1
	(-1.6, 0.2)	(-2.4, 0.1)	**(0.5, 3.7)**	(-5.1, -0.4)	(-2.8, 0.6)	(-3.0, 0.8)	(-2.8, 0.5)	(-0.3, 0.2)
**Dry**								
**PM_1**	-4.3 ***	-2.7 *	**-3.7** **	-3.3	**-7.5** ***	**-8.1** ***	**-7.0** ***	**-0.6** *
	(-6.4, -2.2)	(-5.1, -0.2)	**(-6.3, -1.2)**	(-7.7, 1.4)	**(-11.4, -3.4)**	**(-12.0, -4.1)**	**(-10.7, -3.2)**	**(-1.1, -0.0)**

(c) Taiwan $PM_{2.5}$

	SDNN	RMSSD	LF/HF	HF	LF	VLF	TP	HR
**All**								
**$PM_{2.5}$**	-1.0 ***<br>(-1.6, -0.4)	-0.6<br>(-1.2, 0.1)	1.0 *<br>(0.0, 2.0)	**-1.8** *<br>**(-3.2, -0.4)**	-1.1<br>(-2.2, 0.1)	-1.5 *<br>(-2.6, -0.3)	-1.5 **<br>(-2.5, -0.4)	0.3 ***<br>(0.2, 0.4)
**Season**	-6.5 ***<br>(-8.1, -4.8)	-13.2 ***<br>(-15.3, -11.2)	8.1 ***<br>(4.8, 11.4)	-17.5 ***<br>(-21.3, -13.6)	-10.6 ***<br>(-14.0, -7.1)	-7.9 ***<br>(-11.0, -4.6)	-10.0 ***<br>(-13.0, -7.0)	4.1 ***<br>(3.2, 5.0)
**Summer**								
**$PM_{2.5}$**	-1.8 ***<br>(-2.7, -1.0)	-1.3 **<br>(-2.2, -0.3)	1.6 *<br>(0.1, 3.1)	-4.0 ***<br>(-5.9, -2.0)	-2.8 **<br>(-4.5, -1.0)	-2.6 **<br>(-4.3, -0.9)	-2.7 ***<br>(-4.3, -1.1)	0.4 ***<br>(0.2, 0.5)
**Winter**								
**$PM_{2.5}$**	-0.5<br>(-1.3, 0.3)	0.1<br>(-0.8, 0.9)	0.5<br>(-0.9, 1.8)	-0.1<br>(-2.0, 1.8)	0.3<br>(-1.3, 2.0)	-1.0<br>(-2.5, 0.6)	-0.6<br>(-2.1, 0.9)	0.3 ***<br>(0.1, 0.4)

(d) Taiwan PM_1

	SDNN	RMSSD	LF/HF	HF	LF	VLF	TP	HR
**All**								
**PM_1**	-1.2 ***<br>(-2.0, -0.5)	-1.0 *<br>(-1.8, -0.2)	1.6 *<br>(0.3, 2.9)	-2.5 **<br>(-4.3, -0.7)	-1.1<br>(-2.7, 0.4)	-1.7 *<br>(-3.2, -0.3)	-1.8 *<br>(-3.1, -0.4)	0.5 ***<br>(0.3, 0.6)
**Season**	-6.3 ***<br>(-8.0, -4.6)	-12.9 ***<br>(-15.0, -10.7)	7.5 ***<br>(4.1, 10.9)	-17.0 ***<br>(-20.9, -12.9)	-10.6 ***<br>(-14.1, -7.0)	-7.7 ***<br>(-11.0, -4.4)	-9.8 ***<br>(-12.9, -6.7)	3.9 ***<br>(3.0, 4.8)
**Summer**								
**PM_1**	-2.5 ***<br>(-3.8, -1.1)	-2.1 **<br>(-3.6, -0.6)	4.0 **<br>(1.5, 6.5)	-6.4 ***<br>(-9.5, -3.2)	-3.2 *<br>(-6.0, -0.3)	-3.2 *<br>(-5.9, -0.4)	**-3.5** **<br>**(-6.0, -0.9)**	0.6 ***<br>(0.4, 0.9)
**Winter**								
**PM_1**	-0.7<br>(-1.6, 0.2)	-0.3<br>(-1.3, 0.7)	0.6<br>(-0.9, 2.1)	-0.7<br>(-2.8, 1.5)	-0.0<br>(-1.8, 1.9)	-1.4<br>(-3.1, 0.4)	-1.0<br>(-2.7, 0.6)	0.4 ***<br>(0.2, 0.6)

Note: ***: $p < 0.001$, **: $p < 0.01$, *: $p < 0.05$.

Table S5 Impacts of 5-min (a) $PM_{2.5}$ and (b) PM_1 exposures on the heart rate variability (HRV) indices and heart rate (HR) of Indonesian subjects (n=49) after removing "outdoor" factor from the models. Numbers presented are changes for a 10 μg/m^3 increase in PM; 95% confidence intervals are listed in the parentheses. Bold numbers indicate that the statistically significant levels have changed compared to the results from the original model with "outdoor".

(a) Indonesia $PM_{2.5}$

	SDNN	RMSSD	LF/HF	HF	LF	VLF	TP	HR
**All**								
**$PM_{2.5}$**	-0.1<br>(-0.5, 0.4)	-0.2<br>(-0.8, 0.3)	0.9 *<br>(0.1, 1.6)	-0.7<br>(-1.8, 0.4)	-0.1<br>(-1.0, 0.9)	0.0<br>(-0.9, 1.0)	0.1<br>(-0.8, 0.9)	0.0<br>(-0.1, 0.1)
**Season**	-9.5 ***<br>(-12.3, -6.7)	-9.0 ***<br>(-12.9, -4.9)	3.9<br>(-1.0, 9.0)	-13.3 **<br>(-20.2, -5.9)	-10.1 **<br>(-15.9, -3.9)	-16.1 ***<br>(-21.0, -11.0)	-14.3 ***<br>(-19.1, -9.4)	7.0 ***<br>(5.4, 8.6)
**Wet**								
**$PM_{2.5}$**	0.3<br>(-0.8, 0.3)	-0.1<br>(-0.7, 0.5)	1.0 *<br>(0.1, 1.9)	-0.7<br>(-2.0, 0.6)	-0.3<br>(-1.4, 0.8)	-0.3<br>(-1.4, 0.8)	-0.3<br>(-1.3, 0.6)	0.1<br>(-0.1, 0.2)
**Dry**								
**$PM_{2.5}$**	-0.5<br>(-1.4, 0.5)	-0.9<br>(-1.9, 0.1)	-0.1<br>(-1.5, 1.2)	-1.2<br>(-3.2, 0.9)	-1.0<br>(-2.8, 0.9)	-0.6<br>(-2.5, 1.3)	-0.5<br>(-2.2, 1.3)	-0.0<br>(-0.2, 0.2)

(b) Indonesia PM_1

	SDNN	RMSSD	LF/HF	HF	LF	VLF	TP	HR
**All**								
**PM_1**	-0.2 (-0.9, 0.4)	-0.6 (-1.3, 0.2)	**1.0 (-0.0, 2.0)**	-1.2 (-2.7, 0.3)	-0.5 (-1.8, 0.8)	-0.3 (-1.6, 1.0)	-0.2 (-1.4, 1.0)	-0.1 (-0.3, 0.1)
**Season**	-9.4 *** (-12.2, -6.5)	-8.8 *** (-12.7, -4.6)	3.9 (-1.0, 9.1)	-13.0 ** (-19.9, -5.6)	-9.8 ** (-15.7, -3.6)	-15.9 *** (-20.8, -10.7)	-14.1 *** (-18.9, -9.1)	7.1 *** (5.5, 8.7)
**Wet**								
**PM_1**	-0.5 (-1.2, 0.2)	-0.6 (-1.5, 0.3)	1.4 * (0.2, 2.6)	-1.5 (-3.3, 0.4)	-0.8 (-2.3, 0.7)	-0.8 (-2.3, 0.6)	-0.8 (-2.1, 0.5)	-0.1 (-0.3, 0.2)
**Dry**								
**PM_1**	-0.9 (-2.0, 0.3)	**-1.3** * **(-2.6, 0.0)**	-0.5 (-2.1, 1.2)	-1.8 (-4.4, 0.8)	-2.0 (-4.2, 0.3)	-1.2 (-3.5, 1.1)	-1.1 (-3.2, 1.0)	-0.1 (-0.4, 0.2)

Note: ***: $p < 0.001$, **: $p < 0.01$, *: $p < 0.05$.

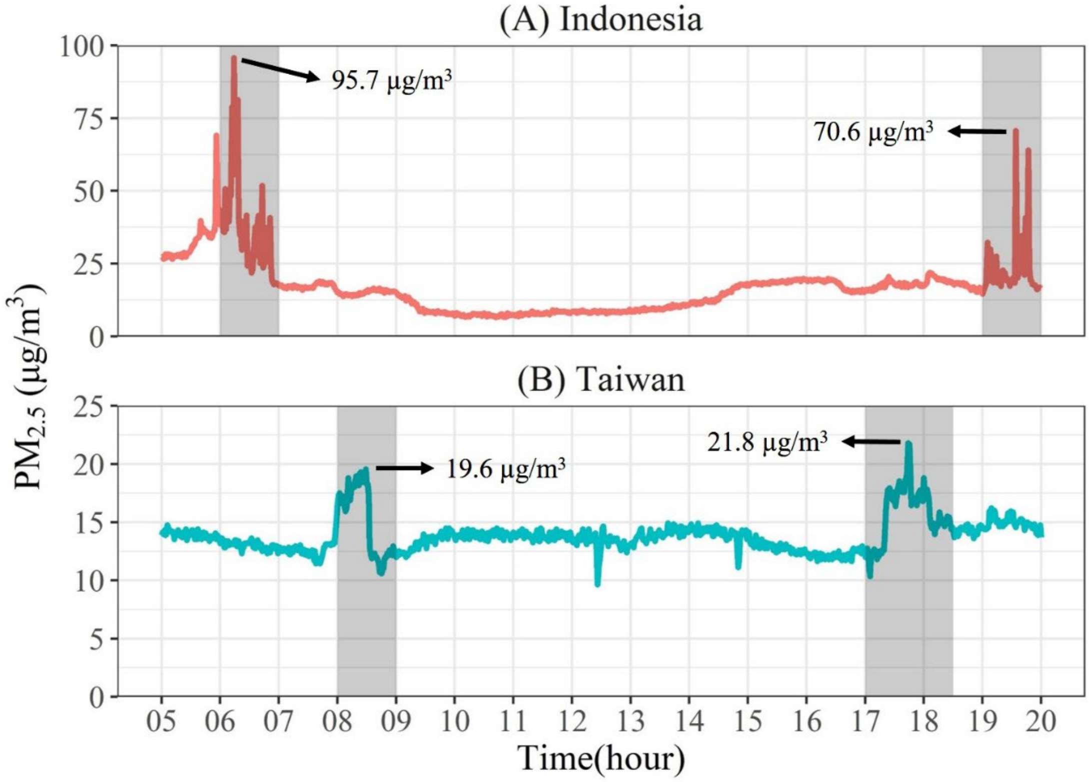

Figure S1 Examples of $PM_{2.5}$ exposures during commute with scooters in (a) Indonesia and (b) Taiwan. During one-hour commuting periods in the morning and evening (gray areas), the highest peak $PM_{2.5}$ for this subject in Indonesia were 95.7 and 70.6 μg/m^3, respectively; while the mean exposure levels were 37.0 ± 16.0 μg/m^3 and 22.9 ± 10.0 μg/m^3, respectively. In Taiwan, the subject was exposed to mean levels of 15.2 ± 3.1 and 15.7 ± 2.4 μg/m^3 in the one-hour morning and evening commuting periods (gray areas), with peak level of 19.6 and 21.8 μg/m^3, respectively.

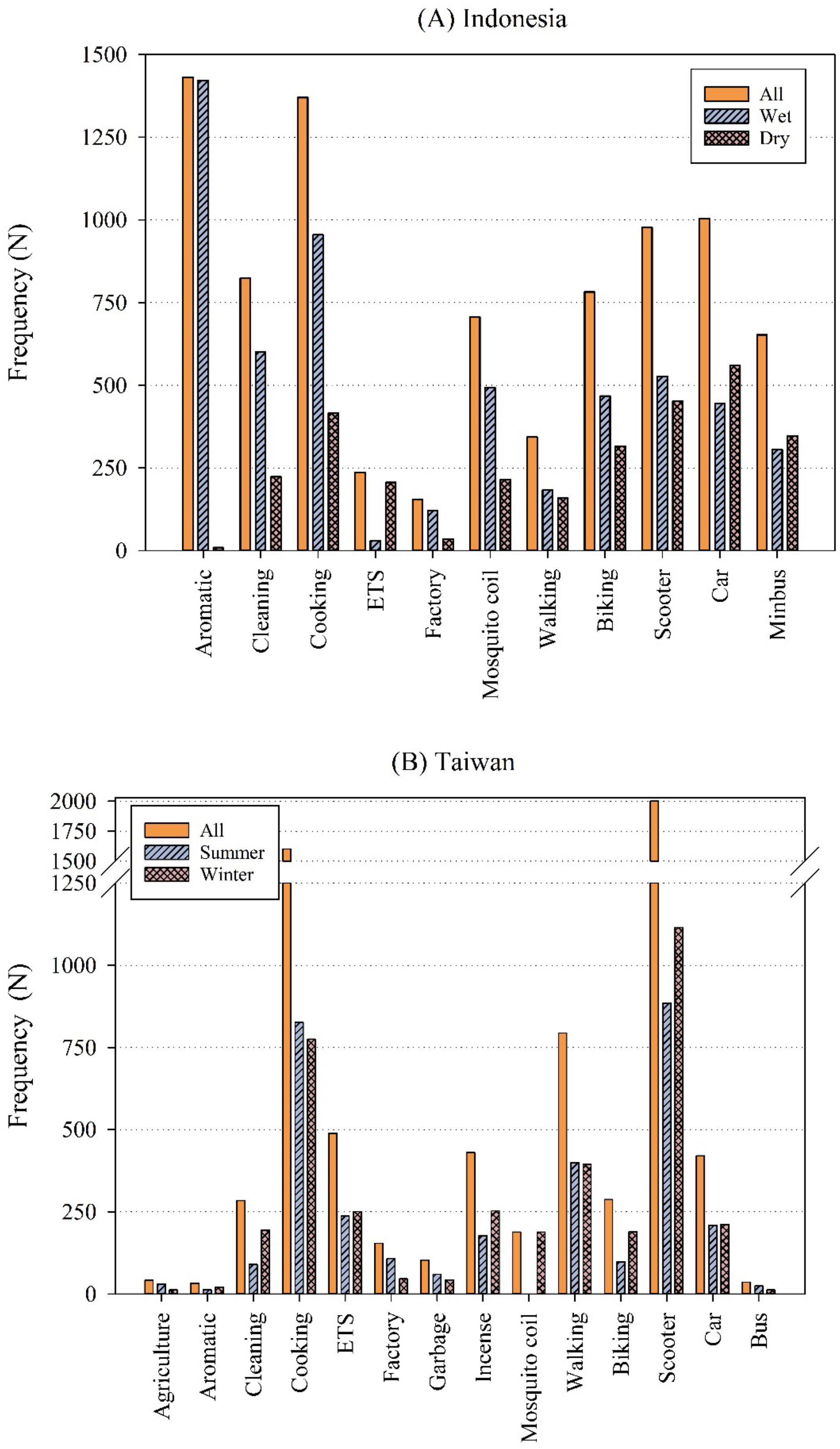

Figure S2 Frequency distribution of various exposure sources or commuting modes (in 5-min resolution) in (a) Indonesia and (b) Taiwan.

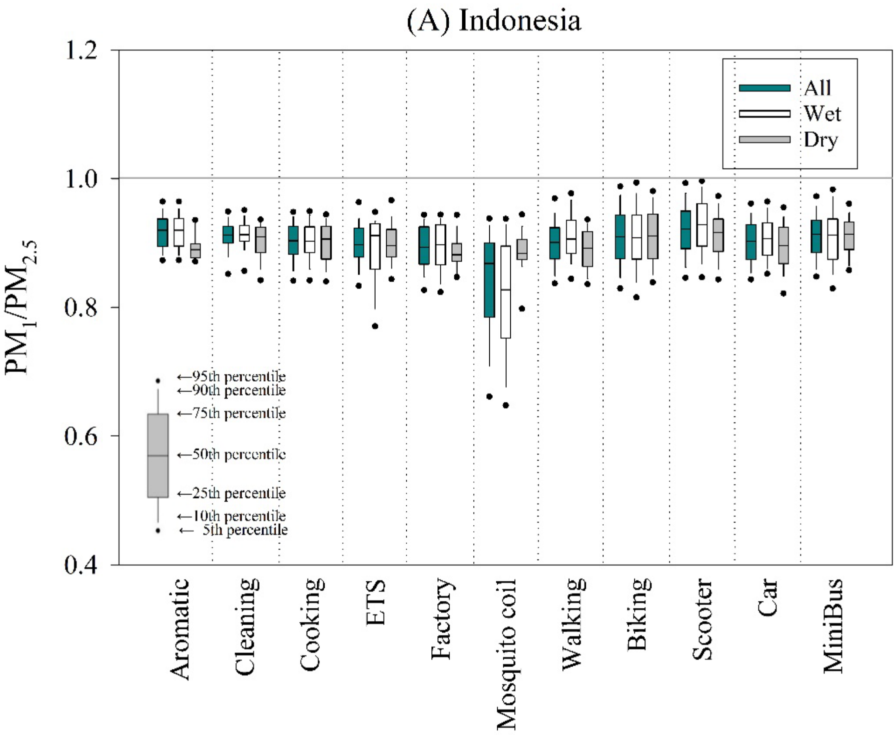

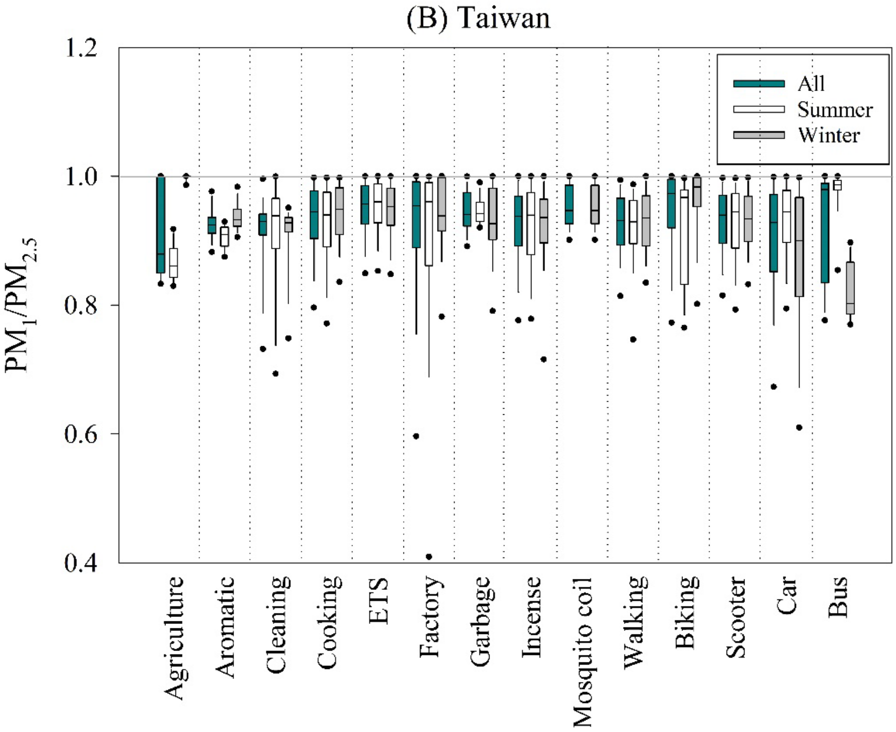

Figure S3 The distribution of 5-min $PM_1/PM_{2.5}$ ratios while exposing to different sources or during different commuting modes in (a) Indonesia and (b) Taiwan.

(a) $PM_{2.5}$

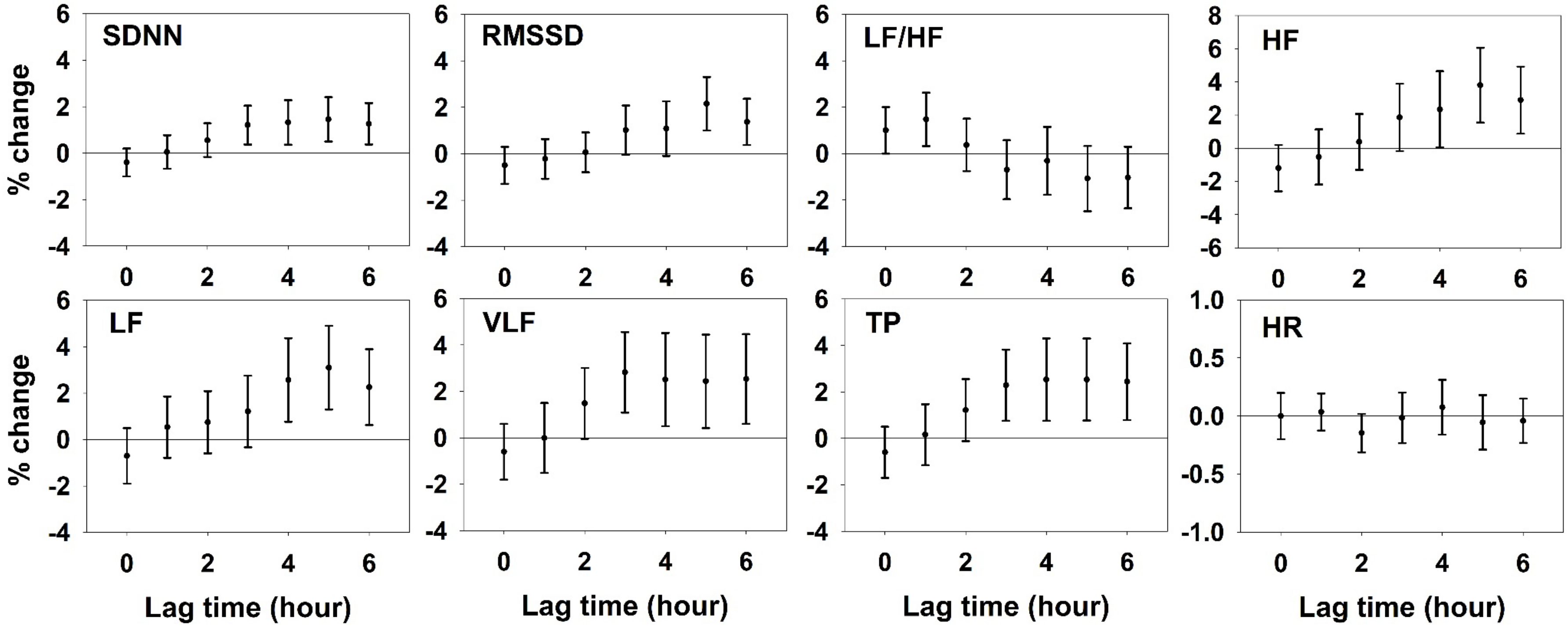

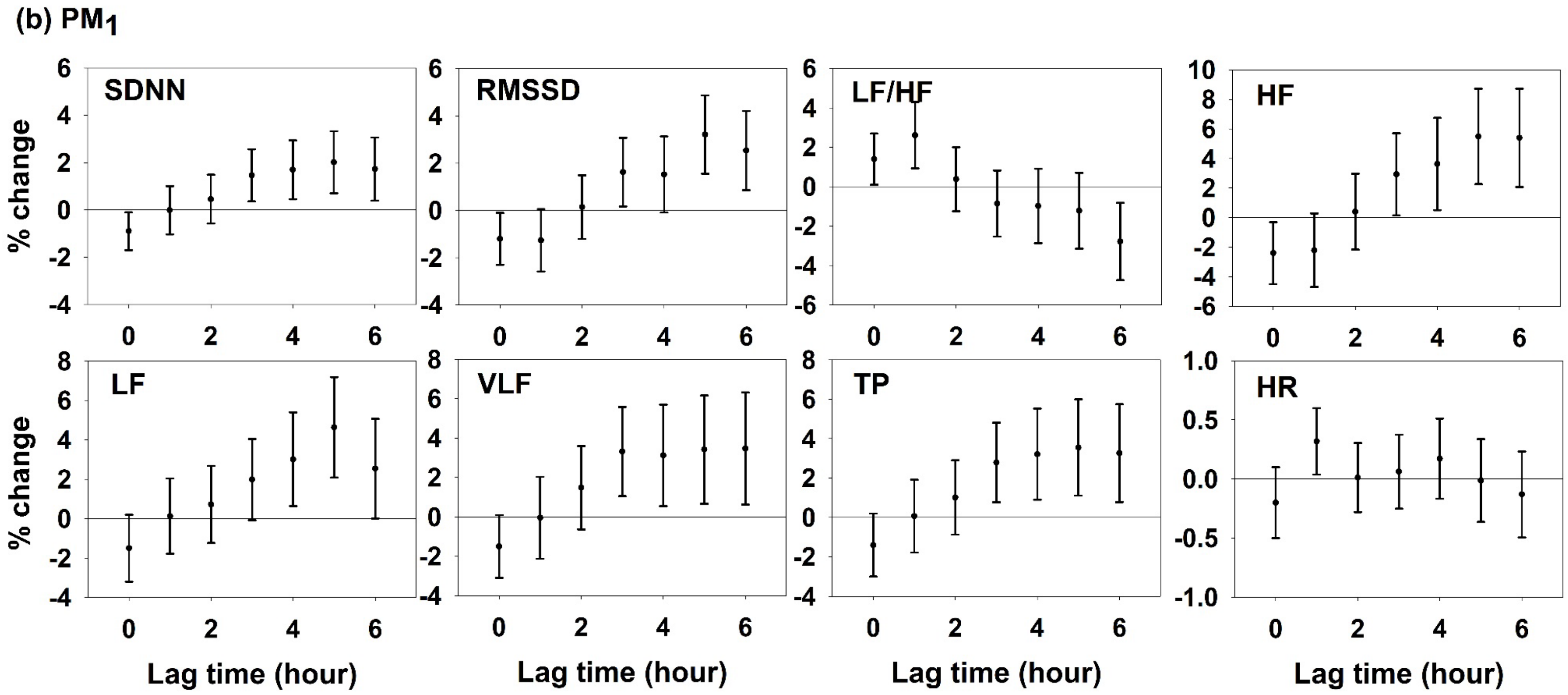

Figure S4 Lag effects of 5-min (a) $PM_{2.5}$ and (b) PM_1 exposures on the HRV indices and HR of the Indonesia scooter group (n=13). Numbers presented are changes for a 10 μg/m^3 increase in PM.

(a) $PM_{2.5}$

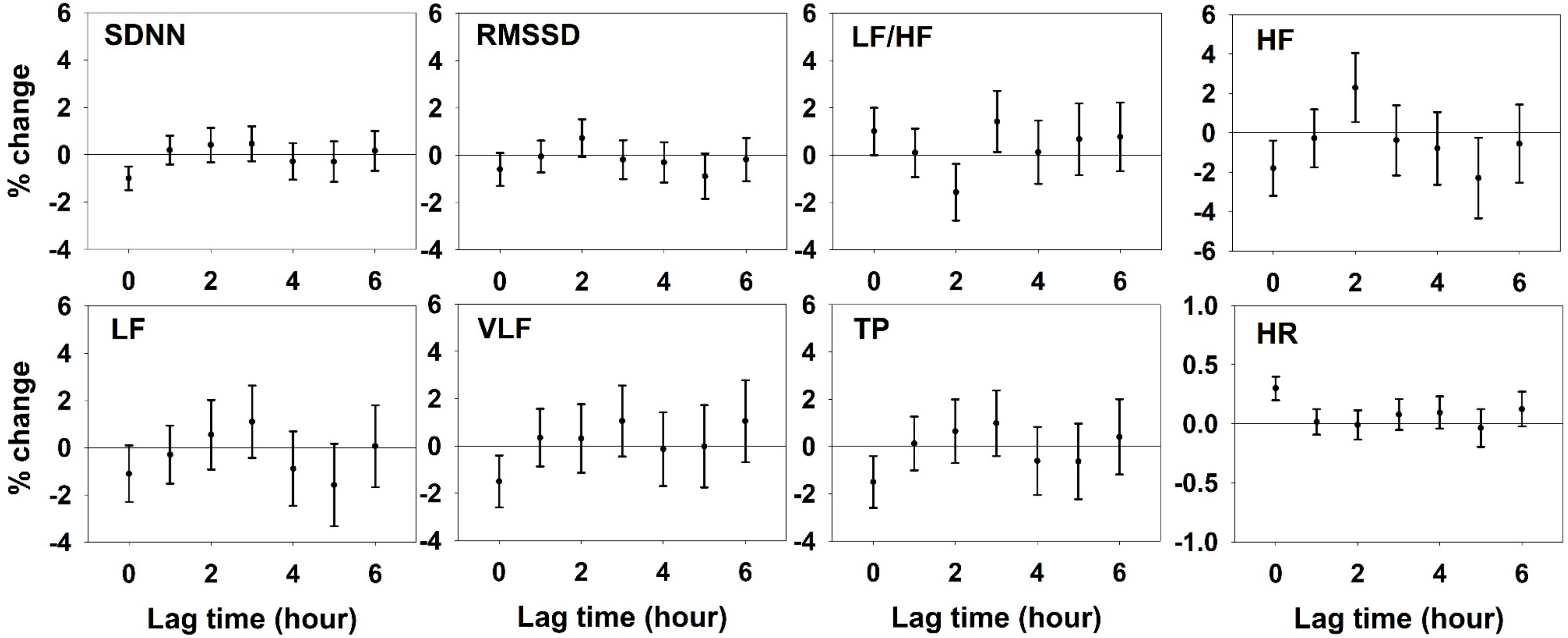
SDNN
RMSSD
LF/HF
HF
LF
VLF
TP
HR
% change
% change
Lag time (hour)
Lag time (hour)
Lag time (hour)
Lag time (hour)

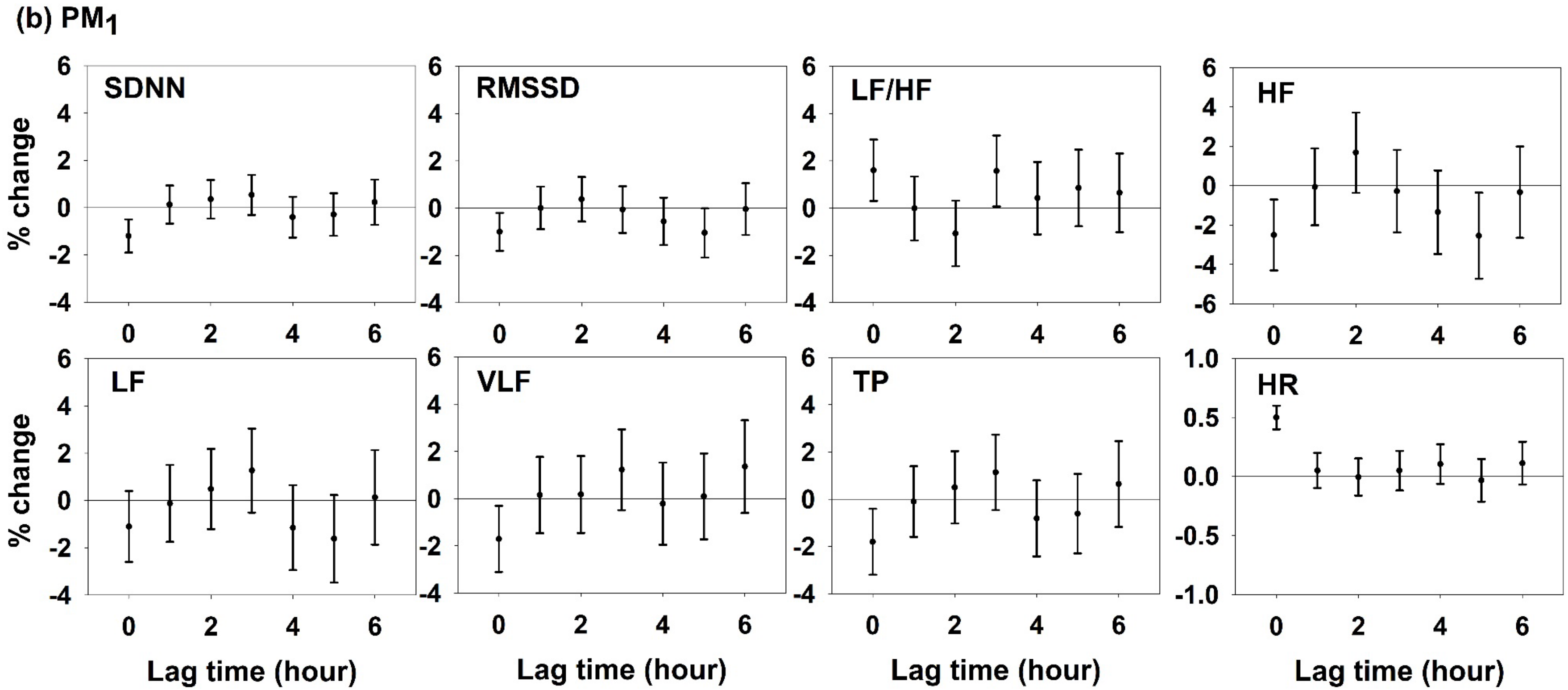

Figure S5 Lag effects of 5-min (a) $PM_{2.5}$ and (b) PM_1 exposures on the HRV indices and HR of all Taiwanese subjects (n=51). Numbers presented are changes for a 10 μg/m^3 increase in PM.
